# Psilocybin: crystal structure solutions enable phase analysis of prior art and recently patented examples

**DOI:** 10.1107/S2053229621013164

**Published:** 2022-01-01

**Authors:** Alexander M. Sherwood, Robert B. Kargbo, Kristi W. Kaylo, Nicholas V. Cozzi, Poncho Meisenheimer, James A. Kaduk

**Affiliations:** a Usona Institute, 2780 Woods Hollow Rd, Madison, WI 53711, USA; b Neuropharmacology Laboratory, University of Wisconsin, School of Medicine and Public Health, 1300 University Avenue, Madison, WI 53706, USA; c Alexander Shulgin Research Institute, 1483 Shulgin Road, Lafayette, CA 94549, USA; d Department of Physics, North Central College, 131 S Loomis Street, Naperville, IL 60540, USA; e Department of Chemistry, Illinois Institute of Technology, 3101 S Dearborn Street, Chicago, IL 60616, USA

**Keywords:** psilocybin, Rietveld, pharmaceutical, psychedelic, qu­anti­tative phase analysis, crystal structure

## Abstract

Psilocybin {systematic name: 3-[2-(di­methyl­amino)­eth­yl]-1*H*-indol-4-yl di­hydro­gen phosphate} is a zwitterionic tryptamine natural product found in numerous species of fungi known for their psychoactive properties. Following its structural elucidation and chemical synthesis in 1959, purified synthetic psilocybin has been evaluated in clinical trials and has shown promise in the treatment of various mental health disorders. In a recent process-scale crystallization investigation, three crystalline forms of psilocybin were repeatedly observed: Hydrate A, Polymorph A, and Polymorph B. The crystal structure for Hydrate A was solved previously by single-crystal X-ray dif­fraction. This article presents new crystal structure solutions for the two anhydrates, Polymorphs A and B, based on Rietveld refinement using laboratory and synchrotron X-ray dif­fraction data, and density functional theory (DFT) calculations. Utilizing the three solved structures, an investigation was conducted *via* Rietveld method (RM) based qu­anti­tative phase analysis (QPA) to estimate the contribution of the three different forms in powder X-ray diffraction (PXRD) patterns provided by different sources of bulk psilocybin produced between 1963 and 2021. Over the last 57 years, each of these samples qu­anti­tatively reflect one or more of the hydrate and anhydrate polymorphs. In addition to qu­anti­tatively evaluating the com­position of each sample, this article evaluates correlations between the crystal forms present, corresponding process methods, sample age, and storage conditions. Furthermore, revision is recommended on characterizations in recently granted patents that include descriptions of crystalline psilocybin inappropriately reported as a single-phase ‘isostructural variant.’ Rietveld refinement demonstrated that the claimed material was com­posed of approximately 81% Polymorph A and 19% Polymorph B, both of which have been identified in historical samples. In this article, we show conclusively that all published data can be explained in terms of three well-defined forms of psilocybin and that no additional forms are needed to explain the diffraction patterns.

## Abstract 2

The crystal structures of anhydrous psilocybin Forms A and B have been solved using laboratory powder X-ray diffraction data, refined using synchrotron and laboratory data, and optimized by applying density functional techniques. The crystal structures, along with that of the previously determined trihydrate, permit the qu­anti­tative analysis of a variety of historical samples of psilocybin.

## Introduction

Psilocybin (see Scheme 1; CASRN 520-52-5) is a psychedelic natural product found in numerous mushrooms around the world. Various species of these mushrooms have been used by humans for thousands of years, especially by people in Mesoamerica, to occasion spiritual or mystical experiences (Schultes *et al.*, 2001 ▸). Psilocybin was first isolated from *Psilocybe* mushrooms, characterized, and later synthesized by Albert Hofmann at Sandoz Ltd (Hofmann *et al.*, 1959 ▸). Throughout the 1960s and 1970s, Sandoz distributed bulk psilocybin and tablets under the trade name Indocybin to researchers and clinicians worldwide. With renewed and increased inter­est in the scientific and clinical study of psy­che­delics (Sherwood & Prisinzano, 2018 ▸; Nichols, 2020 ▸), psilocybin has been evaluated in modern clinical trials aimed at understanding its safety and efficacy in treating a range of mental health conditions, including addiction disorders, depression, and anxiety (Griffiths *et al.*, 2006 ▸, 2016 ▸; Bogenschutz *et al.*, 2015 ▸; Barrett *et al.*, 2018 ▸). Recently, some 50 years after Sandoz production, re-evaluation of psilocybin as a pharmaceutical tool has driven efforts such as contemporary commercial-scale production, systematic physical characterization of the crystalline active pharmaceutical ingredient (API), as well as attempts to block manufacturing of psilocybin through patent protection. 
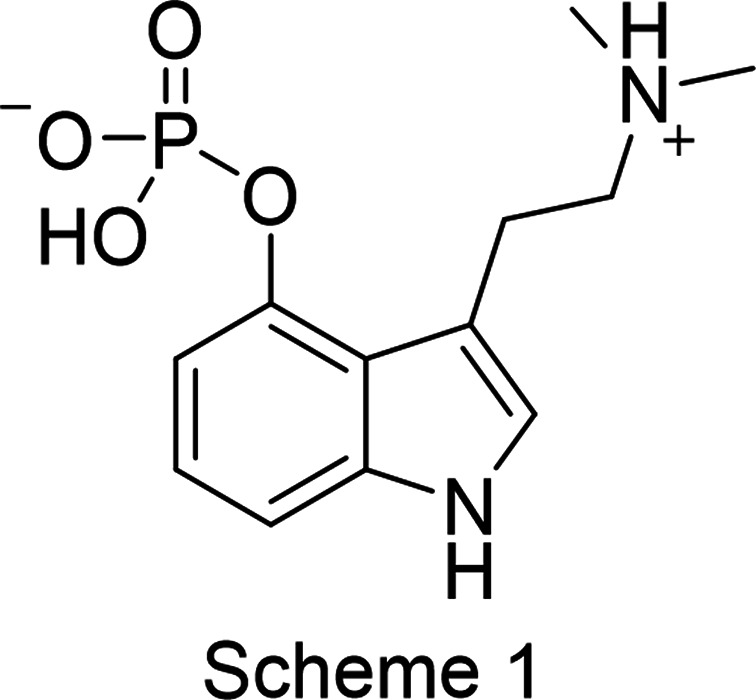


In the decades following the first description of the chemical synthesis of psilocybin and driven in part by the distribution of bulk material by Sandoz, early literature reports on crystallization and solid-state analysis provided evidence for the existence of at least four distinct solid forms: a methanol monosolvate, an ethanol monosolvate, a hydrate, and the corresponding dehydrated/desolvated anhydrate (Baker *et al.*, 1973 ▸; Weber & Petcher, 1974 ▸; Kuhnert-Brandstätter & Heindl, 1976 ▸). The crystal structure for the ethanol solvate was reported by Baker *et al.* (1973 ▸), followed by that for the methanol monosolvate by Weber & Petcher (1974 ▸). Kuhnert-Brandstätter & Heindl (1976 ▸), using IR spectroscopy and differential scanning calorimetry (DSC), showed that both dehydration of a psilocybin hydrate and desolvation of the methanol solvate gave rise to the same anhydrous form rather than two different polymorphs. In the same report, the authors noted that the commercial drug provided by Sandoz was received as the common desolvated/anhydrous form. In addition, the authors described the preparation of the hy­dra­ted form by crystallization from water or from organic solvents with a low water content, which resulted in thin needle-shaped crystals. The authors observed that only by using anhydrous methanol was the methanol solvate formed as unique crystals with a plate morphology and that trace amounts of water favored the formation of the hydrate. Although powder X-ray diffraction (PXRD) was not used, the characterizations in Kuhnert-Brandstätter & Heindl were well correlated with the properties of both Hydrate A and anhydrous Polymorph A, which are described below. A year earlier, Folen (1975 ▸) provided the first known PXRD data on a sample of reagent-grade psilocybin with the intended use in forensic applications, reported as a table of *d*-spacings and intensity values. Though the PXRD apparatus and data collection techniques in Folen were typical for the time, the resolution and accuracy of the data was sufficient to enable modern inter­pretation and is elaborated upon in §4.1.

**Figure 1:**
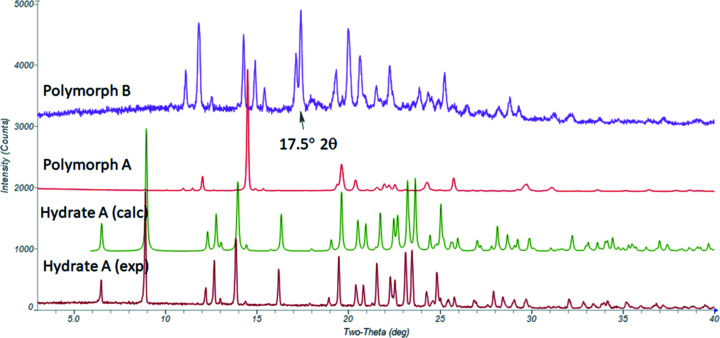
PXRD patterns for psilocybin Hydrate A, showing the experimental, calculated from OKOKAD, Polymorph A, and Polymorph B with the 17.5° 2θ reflection annotated (Cu *K*α radiation).

**Figure 2:**
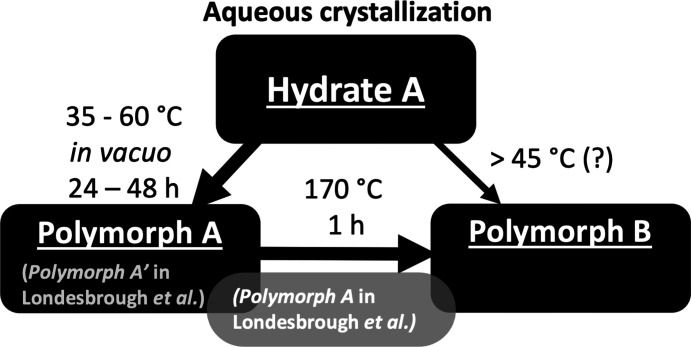
Inter­conversion relationships between psilocybin Hydrate A, Polymorph A, and Polymorph B com­pared to *Polymorph A′* and *Polymorph A* described in Londesbrough *et al.* (2019 ▸), where *Polymorph A* (gray box) is a mixture of both Polymorph A and Polymorph B.

More recently, synthetic process development efforts (Kargbo *et al.*, 2020 ▸; Londesbrough *et al.*, 2019 ▸) provided additional systematic characterization of Hydrate A and the corresponding anhydrate Polymorph A by PXRD (Fig. 1 ▸). These hydrate and anhydrate forms are expected to have been originally observed by Kuhnert-Brandstätter and Heindl, given the consistent DSC thermograms and analogous preparation conditions using aqueous crystallization to give the hydrate followed by vacuum drying to the anhydrous form (Fig. 2 ▸). The crystal structure of Hydrate A was subsequently solved by single-crystal X-ray diffraction [Arlin *et al.*, 2019 ▸; Cambridge Structural Database (CSD; Groom *et al.*, 2016 ▸) refcode OKOKAD]. The hydrate crystallizes in the ortho­rhom­bic space group *Pbca* as a trihydrate. Subsequent investigation *via* large-scale crystallization studies resulted in the identification of an additional PXRD pattern that was de­scribed as Polymorph B (*vida infra*), an anhydrate formed by briefly heating Polymorph A at 160 °C. Experiments described in §4.1 also provided new evidence to suggest a mechanism by which Polymorph B was formed directly from Hydrate A at atmospheric pressure at tem­per­atures in the range 45–55 °C, as depicted in Fig. 2 ▸.

A Compass Pathways patent application (Londesbrough *et al.*, 2019 ▸) describes crystalline psilocybin characterized by PXRD reflections that are consistent with the anhydrate Polymorph A (as depicted herein) and is denoted in the patent application as *Polymorph A′* [*i.e.* A-prime; italics used throughout to distinguish crystal form descriptions used in Londesbrough *et al.* (2019 ▸) from those used herein; see Fig. 2 ▸ for clarification]. A second material described by a similar powder diffraction pattern is denoted in the patent application as psilocybin *Polymorph A*. According to the application, ‘[a] peak at about 17.5 ± 0.1° 2θ distinguishes psilocybin *Polymorph A* from *Polymorph A′*.’ The application further describes *Polymorph A′* as an ‘isostructural variant’ of *Polymorph A*, where the perturbation at 17.5° 2θ was, ‘more pronounced for psilocybin produced at large scale (com­pared to that obtained at small scale) and was unexpected.’ The application also asserts that, ‘*Polymorph A* is the true form with *Polymorph A′* formed at small scale being atypical.’ The terms ‘isostructural variant’ and ‘true form’ were not elaborated upon and appear ambiguous. According to the IUCr online dictionary of crystallography, the term ‘isostructural’ describes two crystals with, ‘the same structure, but not necessarily the same cell dimensions nor the same chemical com­position, and with a ‘com­parable’ variability in the atomic coordinates to that of the cell dimensions and chemical com­position.’ Considering the accepted definitions of isostructural and variant, the authors’ combination of these terms did not assist in characterizing crystalline psilocybin samples showing minor variation in the appearance of their respective PXRD patterns. The patent application also provided a PXRD pattern for the second anhydrous form, Polymorph B (consistent with Polymorph B herein), which was produced by heating *Polymorph A* to 160 °C. However, the authors of the patent neglected to address the possibility that the material described as *Polymorph A* (as labeled in the patent application), with the weak 17.5° 2θ PXRD reflection, consisted of a mixture of their *Polymorph A′* and Polymorph B and instead noted only the absence of Hydrate A reflections in the PXRD pattern.

A known challenge in process-scale API isolation is heterogeneous heating during the final isolation step with vacuum drying (Ottoboni *et al.*, 2020 ▸; Airaksinen *et al.*, 2004 ▸; Lim *et al.*, 2016 ▸). Taken together with the dynamics of large-scale API drying, the observation of a prominent reflection at 17.5° 2θ in the diffractogram for anhydrate Polymorph B (Fig. 1 ▸) and the reported thermal inter­conversion behavior between Polymorph A and Polymorph B provided foundation for the following hypothesis: *Polymorph A* in Londesbrough *et al.* (2019 ▸), which was isolated only from large-scale batches, and distinguished from *Polymorph A′* by an additional weak reflection at 17.5° 2θ in the PXRD pattern, was not an ‘isostructural variant,’ but instead was a mixture of the anhydrous Polymorph A and Polymorph B (Fig. 2 ▸) resulting from inadequately controlled drying at large scale. The observed discrepancy in the diffractograms for the two materials was far more characteristic of a mixture of the two phases rather than an inherently novel single-phase crystalline form.

Given the pre­ponderance of literature examples describing the crystallization of psilocybin from water or methanol with subsequent drying to a constant weight under vacuum with heat (Hofmann *et al.*, 1959 ▸, 1965 ▸; Heim *et al.*, 1965 ▸; Kuhnert-Brandstätter & Heindl, 1976 ▸; Nichols & Frescas, 1999 ▸; Londesbrough *et al.*, 2019 ▸; Kargbo *et al.*, 2020 ▸; Sherwood *et al.*, 2020 ▸), we envisaged that a review of PXRD analysis on samples of bulk psilocybin produced in the years following Hoffman’s first reported 1959 synthesis and crystallization would reveal results consistent with the presence of Hydrate A, Polymorph A, and/or Polymorph B phases in varying proportions, and that the so-called ‘isostructural variant,’ *Polymorph A* described in Londesbrough *et al.* (2019 ▸) would be exposed as a mixture of Polymorph A and Polymorph B, and that the phase identified as *Polymorph A′* was really Polymorph A described herein.

To support an investigation into the identification and qu­anti­fication of crystalline phases present in bulk psilocybin samples, qu­anti­tative phase analysis (QPA) by the Rietveld Method (RM) was identified as a viable approach. The technique relies on fitting an experimental diffraction pattern from a suspected multiphase sample with a calculated profile based on the crystal structures for each of the phases. The calculated model considers the sum of the individual crystal structure parameters, unit-cell dimensions, peak shapes, widths, backgrounds, and preferred orientation effects (Hill, 1991 ▸). To com­plete this work, crystal structure models were therefore required for psilocybin Hydrate A, Polymorph A, and Polymorph B.

Hydrate A provided single crystals suitable for structure solution as described in Arlin *et al.* (2019 ▸), which were used in the QPA study. Although pure Polymorph A or Polymorph B could be isolated from drying and heating Hydrate A, the concomitant collapse of the hydrate on drying to Polymorph A was found to induce fracturing of the solid, resulting in crystals of Polymorph A that were too small to evaluate the structure *via* single-crystal X-ray diffraction. Similarly, prolonged heating of Polymorph A to provide Polymorph B did not provide suitable single crystals. Additionally, no solvent-based processes were identified that provided single-phase Polymorph A or Polymorph B directly, and so they were only able to be isolated from drying and heating Hydrate A. Therefore, structure solutions for Polymorphs A and B necessitated indexing of the available powder patterns from both laboratory and synchrotron data, allowing solution of the crystal structures using Monte Carlo simulated annealing techniques, as described in the *Experimental* section. Based on the structure models for Hydrate A, Polymorph A, and Polymorph B, QPA by RM was used to estimate the relative amounts of the three forms that were present in the PXRD patterns depicted in Londesbrough *et al.* (2019 ▸) and in PXRD patterns from bulk psilocybin samples of various origins produced between 1963 and 2021.

## Experimental

### General preparation of psilocybin Hydrate A, Polymorph A, and Polymorph B

Bulk psilocybin was manufactured according to Kargbo *et al.* (2020 ▸). Briefly, Hydrate A was isolated from crystallization in water and provided the representative diffractogram consistent with the predicted pattern for the solved crystal structure in Arlin *et al.* (2019 ▸) (Fig. 1 ▸). Alternatively, Hydrate A was prepared by recrystallization of psilocybin from 30% aqueous acetone (100 ml g^−1^) as described in Sherwood *et al.* (2020 ▸). Hydrate A was converted to Polymorph A by drying the solid at 35–45 °C under vacuum for at least 24 h and sub­se­quently at 50–60 °C under vacuum for at least 24 h. The process was carried out on a 1.2 kg scale, as described in Kargbo *et al.* (2020 ▸). Thermal cycling of a sample of Polymorph A to 160 °C for 1 h under a steady stream of nitro­gen provided the PXRD pattern for Polymorph B.

### Structure solutions for Polymorph A and Polymorph B (Table 1 ▸)

Pattern CG-0026-001-01.asc (Polymorph A, the same manufacturing lot as Sample 19 and provided by conditions described in §2.1) was indexed using *JADE Pro* (MDI, 2021 ▸) on a primitive ortho­rhom­bic unit cell having *a* = 17.40109, *b* = 16.00553, *c* = 9.33183 Å, *V* = 2599.04 Å^3^, and *Z* = 8. The suggested space group was *Pbca*, which was confirmed by successful solution and refinement of the structure. A reduced cell search in the CSD yielded 90 hits, but limiting the chemistry to C, H, N, O, and P atoms resulted in no hits.

A psilocybin mol­ecule was extracted from the OKOKAD structure using *Materials Studio* (Dassault Systèmes, 2021 ▸), and saved as a .mol2 file. The structure was solved using Monte Carlo simulated annealing techniques as implemented in *EXPO2014* (Altomare *et al.*, 2013 ▸).

To facilitate refinement of the Polymorph A structure, four samples of psilocybin were examined using synchrotron radiation. Polymorph A samples were crystallized according to the methods described in §2.1 and were held in a vacuum oven at 30–40 °C immediately prior to preparation for analysis. The white powders were packed into 1.5 mm diameter Kapton capillaries and rotated during the measurements at ∼50 Hz. The powder patterns were measured at 295 K at beamline 11-BM (Lee *et al.*, 2008 ▸; Wang *et al.*, 2008 ▸; Antao *et al.*, 2008 ▸) of the Advanced Photon Source at Argonne National Laboratory using a wavelength of 0.458162 (2) Å in the range 0.5–50° 2θ with a step size of 0.001° and a counting time of 0.1 s per step. The high-resolution powder diffraction data were collected using 12 silicon crystal analyzers that allow for high angular resolution, high precision, and accurate peak positions. A silicon (NIST SRM 640c) and alumina (SRM 676a) standard (ratio Al_2_O_3_:Si = 2:1 by weight) was used to calibrate the instrument and refine the monochromatic wavelength used in the experiment. Two of the samples contained about 0.7 wt% of the trihydrate, and two were phase pure. All four refined Polymorph A structures were essentially identical, so only one will be discussed here.

Rietveld refinement was carried out using *GSAS-II* (Toby & Von Dreele, 2013 ▸). Only the 2.0–30.0° portion of the pattern was included in the refinement (*d*_min_ = 0.885 Å). All non-hydrogen-bond distances and angles were subjected to restraints, based on a *Mercury*/*Mogul* Geometry Check (Sykes *et al.*, 2011 ▸; Bruno *et al.*, 2004 ▸). The *Mogul* average and standard deviation for each qu­antity were used as the restraint parameters. The restraints contributed 1.3% to the final χ^2^. The H atoms were included in calculated positions, which were recalculated during the refinement using *Materials Studio* (Dassault Systèmes, 2021 ▸). The *U*_iso_ values were grouped by chemical similarity; the *U*_iso_ values for the H atoms were fixed at 1.3 times the *U*_iso_ values of the heavy atoms to which they are attached. The peak profiles were described using the generalized microstrain model. The background was modeled using a 9-term shifted Chebyshev polynomial. A 4th-order spherical harmonic model was used to model the preferred orientation; the texture index was 1.133 (1).

**Figure 3:**
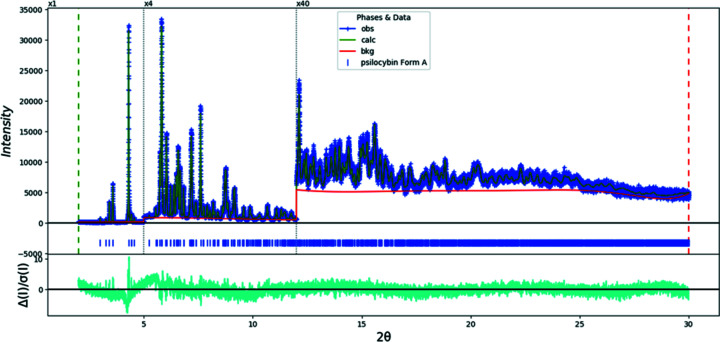
The Rietveld plot for the refinement of psilocybin Form A using synchrotron data. The blue crosses represent the observed data points and the green line is the calculated pattern. The cyan curve is the normalized error plot. The vertical scale has been multiplied by a factor of 5 for 2θ > 5.0° and by a factor of 40 for 2θ > 12.0°.

The final refinement of 85 variables using 28045 observations and 49 restraints yielded the residuals *R*_wp_ = 0.0740 and GOF = 1.36. The largest peak (1.17 Å from atom C10 in the center of the aromatic six-membered ring) and hole (1.38 Å from H27) in the difference Fourier map were 0.36 (9) and −0.44 (9) e Å^−3^, respectively. The largest errors in the difference plot (Fig. 3 ▸) are in the shape of the strong 021 peak at 4.30° and in the background.

A density functional geometry optimization was carried out using VASP (Kresse & Furthmüller, 1996 ▸) (fixed experimental unit cell) through the *MedeA* graphical inter­face (Materials Design, 2016 ▸). The calculation was carried out on 16 2.4 GHz processors (each with 4 Gb RAM) of a 64-processor HP Proliant DL580 Generation 7 Linux cluster at North Central College. The calculation used the GGA-PBE functional, a plane wave cutoff energy of 400.0 eV, and a *k*-point spacing of 0.5 Å^−1^, leading to a 1 × 1 × 2 mesh, and took ∼40 h. A single-point density functional calculation (fixed experimental cell) and population analysis were carried out using *CRYSTAL17* (Dovesi *et al.*, 2018 ▸). The basis sets for the H, C, N, and O atoms in the calculation were those of Gatti *et al.* (1994 ▸), and the basis set for P was that of Peintinger *et al.* (2013 ▸). The calculations were run on a 3.5 GHz PC using 8 *k*-points and the B3LYP functional, and took ∼2.6 h.

The pattern Form H_CG-0019E-038-03 + 24hrs.xrdml (Sample 21), representative of Polymorph B, was indexed using *DICVOL14* (Louër & Boultif, 2014 ▸) on a primitive mono­clinic cell having *a* = 10.3584, *b* = 15.4098, *c* = 17.1496 Å, β = 95.248°, *V* = 2725.96 Å^3^, and *Z* = 8. *EXPO2014* suggested the space group *P*2_1_/*a*, which was confirmed by successful solution and refinement of the structure. A reduced cell search in the CSD yielded 30 hits, but no structures for psilocybin deri­vatives. The structure was solved using Monte Carlo simu­lated annealing techniques, as implemented in *EXPO2014*, using two psilocybin mol­ecules as fragments. Two of the ten trials yielded residuals much better than the others and having suitable hydrogen-bonding patterns. Rietveld refinement and density functional theory (DFT) optimization were carried out using similar strategies as for Polymorph A, except that the displacement coefficients were not refined and an isotropic microstrain model was used for the profiles.

**Figure 4:**
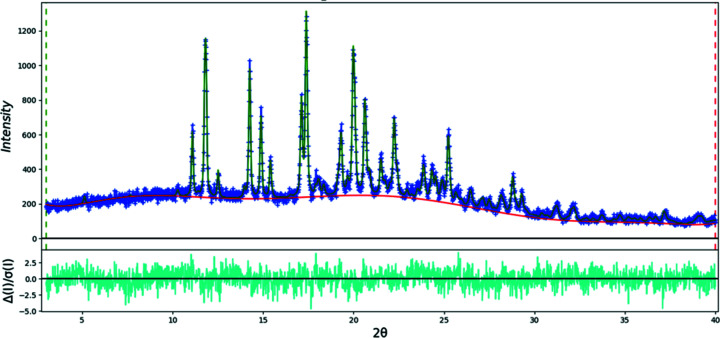
The Rietveld plot for the refinement of psilocybin Form B using laboratory data. The blue crosses represent the observed data points and the green line is the calculated pattern. The cyan curve is the normalized error plot.

The final refinement of 130 variables using 2868 observations and 98 restraints yielded the residuals *R*_wp_ = 0.0749 and GOF = 1.23. The largest peak (1.63 Å from O2) and hole (1.54 Å from C67) in the difference Fourier map were 0.14 (4) and −0.17 (4) e Å^−3^, respectively. The difference plot (Fig. 4 ▸) is quite flat.

DFT optimization and population analysis were carried out for the trihydrate OKOKAD structure (Arlin *et al.*, 2019 ▸) using similar procedures. Calculations were carried out using the fixed 152 K single-crystal and 295 K powder cells. A dis­persion-corrected DFT calculation was also carried out (using the DFT-D3 model for van der Waals inter­actions), starting from the room-tem­per­ature cell.

### Psilocybin samples – sources and preparation

In total, PXRD analysis was obtained for 24 unique samples of psilocybin from multiple sources (Table 2 ▸). Individual sample names were assigned a sample code (Samples 1–24), sorted by ascending date of availability, and classified as commercially available, literature sourced, clinical trial materials, reported in the patent literature, or produced during process development. Individual sample descriptions with crystallization conditions (where known) are reported in §2.3.1–9. Powder diffraction data were collected from multiple sources and the corresponding diffractometer parameters are also reported in §2.3.1–9.

#### RTI-1823-17-15 (1)

Sample RTI-1823-17-15 (1) was provided by the National Institute on Drug Abuse–Drug Supply Program (NIDA–DSP). The sample was obtained from an archived lot of psilocybin originally produced by Sandoz Ltd (Basel, Switzerland), Batch 944369, dated December 1963 (see Fig. S1 in the supporting information for an image of the container). Exact recrystallization parameters for this lot were not available; however, Hofmann *et al.* (1959 ▸, 1965 ▸) reported that samples from the same era were recrystallized from either water or methanol and further dried to a constant weight. PXRD analysis for this sample was performed on a Bruker D8 Advance diffractometer operating in reflection-mode geom­etry with a LynxEye detector and monochromatic incident radiation of wavelength 1.54056 Å scanned in the range 2–50° 2θ, with a 17.5 min data collection time.

#### Folen (2)

PXRD data for this psilocybin sample were published in Folen (1975 ▸). The sample source was described as reagent grade and crystallization conditions for the sample were not available. The PXRD data were provided as a table of *d*-spacing values and corresponding relative intensities based on monochromatic incident radiation of wavelength 1.54056 Å and were acquired by manual analysis of the strip chart recording.

#### USP 0274-F (3)

Sample USP 0274-F (3) was a gift from Dr Paul Daley of the Alexander Shulgin Research Institute. This sample was a USP (Rockville, MD) Authentic Substance, Psilocybin code 0274-F, Lot 216420301 (see Fig. S2 in the supporting information for an image of the container). The container states that the material ‘contains about 0.8% moisture.’ United States Pharmacopeial Convention was unable to provide a precise date of manufacture as records are not made public for Authentic Substances. A record-of-use log from the original owner (Dr Alexander Shulgin) shows it was used as early as 1976 and this date was used in Table 2 ▸. PXRD analysis was carried out using the same conditions outlined for Sample 1.

#### 10415-25 (4)

Sample 10415-25 (4) was provided by the Johns Hopkins University School of Medicine clinical pharmacy. The psilocybin was originally synthesized by Dr David Nichols (Purdue University, Lafayette, IN, USA) and distributed to Johns Hopkins University and the University of New Mexico for use in human clinical trials. According to the batch record, this material was produced by recrystallization from boiling H_2_O (10 ml H_2_O per gram of psilocybin). After drying under high vacuum at 60 °C, the anhydrous material had a melting point of 219–220 °C. PXRD analysis was performed on a Rigaku Smart-Lab diffractometer operating in either transmission or reflection geometry with a D/teX Ultra detector and monochromatic incident radiation of wavelength 1.54056 Å. Transmission geometry samples were scanned in the range 3–40° 2θ with a 20 min data collection time. Re­flec­tion geometry samples were scanned in the range 2–40° 2θ with a 6 min data collection time. This lot of psilocybin supported several clinical trials (Bogenschutz *et al.*, 2015 ▸; Barrett *et al.*, 2018 ▸; Griffiths *et al.*, 2006 ▸, 2016 ▸).

#### Ψ-67-2, Ψ-81-1, and Ψ-97-1 (5–7)

Entries 5–7 were synthesized by author Nicholas V. Cozzi at the University of Wisconsin–Madison. Crude psilocybin from these syntheses was recrystallized by dissolving the material in boiling H_2_O (10 ml H_2_O per gram of psilocybin), then allowing the solution to stand for 12 h at 4 °C. The resulting crystals were harvested by suction filtration, washed with ice-cold water, then dried under high vacuum. Batch Ψ-67-2, synthesized in April 2013 (Table 2 ▸, entry 5), was dried in a vacuum oven for 1 h at 40 °C. This batch was used for process development. Batches Ψ-81-1 and Ψ-97-1 (Table 2 ▸, entries 6 and 7) were both synthesized in July 2013 and were dried in a vacuum oven at 50 °C for 7 and 5 h, respectively. These batches were employed in published clinical trials (Brown *et al.*, 2017 ▸; Nicholas *et al.*, 2018 ▸). PXRD analysis was carried out on these samples using the same conditions outlined for Sample 1.

#### *Polymorph A*, *Polymorph A’*, Polymorph B, and Hydrate A (8–11)

Diffractograms and analysis parameters for Compass Pathways’ *Polymorph A* (8) and *Polymorph A′* (9), Polymorph B (10), and Hydrate A (11) were reported in Londesbrough *et al.* (2019 ▸) [patent Figs. 7(*a*), 7(*b*), 7(*c*), and 7(*d*), respectively], and the corresponding crystallization conditions were described. Briefly, Samples 8 and 11 (*Polymorph A* and Hydrate A as denoted in the patent) were produced by recrystallizing crude psilocybin (94 g) from water (9.6 ml per gram of psilocybin). The heated aqueous solution was cooled and seeded with Hydrate A crystals and the resulting solid was isolated by vacuum filtration to provide Hydrate A, which was dried under vacuum at 50 °C for 30 h. Preparation of Sample 9, *Polymorph A′* (as denoted in the patent), was analogous to that of Sample 8, except that the crystallization was conducted using 1.0 g of crude psilocybin with 12.8 ml of water, and the collected solid was dried under vacuum at 50 °C for 16 h. Finally, Sample 10, Polymorph B, was prepared by heating psilocybin Polymorph A to 173 °C for 5 min.

#### SPS5107/20/1, 17/44/136G, 17/44/132E, 17/44/116Z, 17/44/123L, 800325750, and 800326600 (12–18)

Sam­ples 12–18 were provided by Usona Institute and were obtained from batches of psilocybin produced during chemistry process development. Samples were recrystallized from aqueous acetone or pure water as reported in Sherwood *et al.* (2020 ▸) and dried to a constant weight at different tem­per­atures and times under the conditions listed in Table 3 ▸. PXRD analysis was performed on a Bruker D8 Advance Eco dif­fractometer operating in reflection-mode geometry with an SSD160 detector and monochromatic incident radiation of wavelength 1.54056 Å scanned in the range 2–45° 2θ with a 3.9 min data collection time.

#### ARN-19-002654, CG002E-004-01, and CG-0019E-038-03 (19–21)

Entries 19–21 were provided by Usona Institute and were obtained from batches of psilocybin pro­duced during chemistry process development by the methods out­lined in Kargbo *et al.* (2020 ▸). Sample 19 provided the reference diffractogram for Polymorph A and was aliquoted from a 1.2 kg scale crystallization. Sample 20 provided the reference diffractogram for Hydrate A and Sample 21 provided the reference diffractogram for Polymorph B. Crystallization conditions are described in §2.1. PXRD analysis was performed on a PANalytical X’Pert Pro diffractometer with a Pixcel detector and monochromatic incident radiation of wavelength 1.54056 Å scanned in the range 3–40° 2θ with an 11 min data collection time. Samples were analysed in transmission mode and held between low-density polyethyl­ene films.

#### PL005E-004-40C, PL005E-004-45C, and PL005E-004-55C (22–24)

Entries 22–24 were provided by Usona Institute and were produced by the thermal stressing of a sample of Hydrate A. Sample 22 was held at 40 °C for 25 h, Sample 23 was held at 45 °C for 25 h, and Sample 24 was held at 55 °C for 25 h. PXRD analysis was carried out using the same conditions outlined for Samples 19–21.

### PXRD pattern fitting by RM

With the solved structures for Hydrate A, Polymorph A, and Polymorph B, the Rietveld refinements were performed using diffractograms from Samples 1–24. Fixed structural models were imported into *GSAS-II*. The Folen peak list was converted (using FWHM = 0.3°, to account for potential variation in peak positions) into a pseudo-raw data file using *Endeavour* (Crystal Impact, 2021 ▸). The patterns from Londesbrough *et al.* (2019 ▸) were digitized using *UN-SCANT-IT* (Silk Scientific, 2013 ▸) and converted to .mdi files using *JADE Pro* (MDI, 2021 ▸), and to *GSAS*-format files using *PowDLL* (Kourkoumelis, 2013 ▸). All of these data sets exhibited significant preferred orientation, which was modeled using the minimum number of spherical harmonics to achieve an acceptable fit.

## Results

### Structure and mol­ecular conformations for Hydrate A, Polymorph A, and Polymorph B

**Figure 5:**
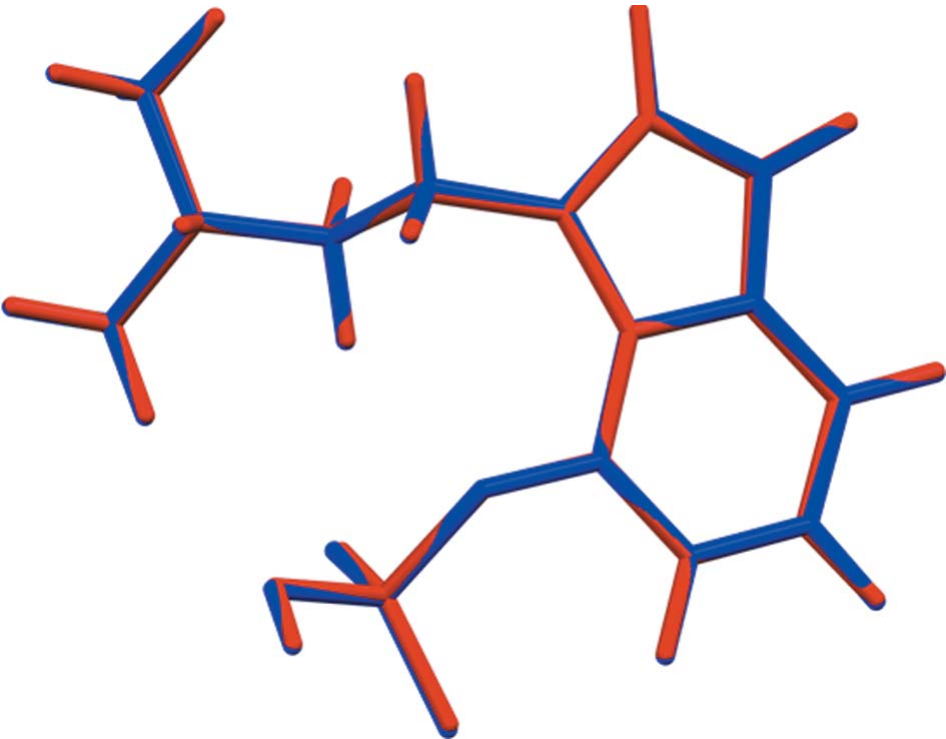
Comparison of the Rietveld-refined (red) and VASP-optimized (blue) structures of psilocybin Polymorph A. The r.m.s. Cartesian displacement is 0.053 Å.

**Figure 6:**
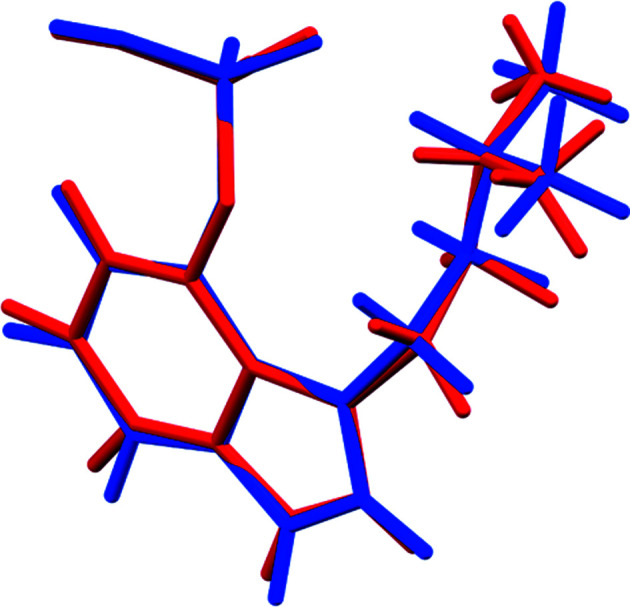
Comparison of the Rietveld-refined (red) and VASP-optimized (blue) structures of psilocybin Polymorph B Mol­ecule 1. The r.m.s. Cartesian displacement is 0.160 Å.

**Figure 7:**
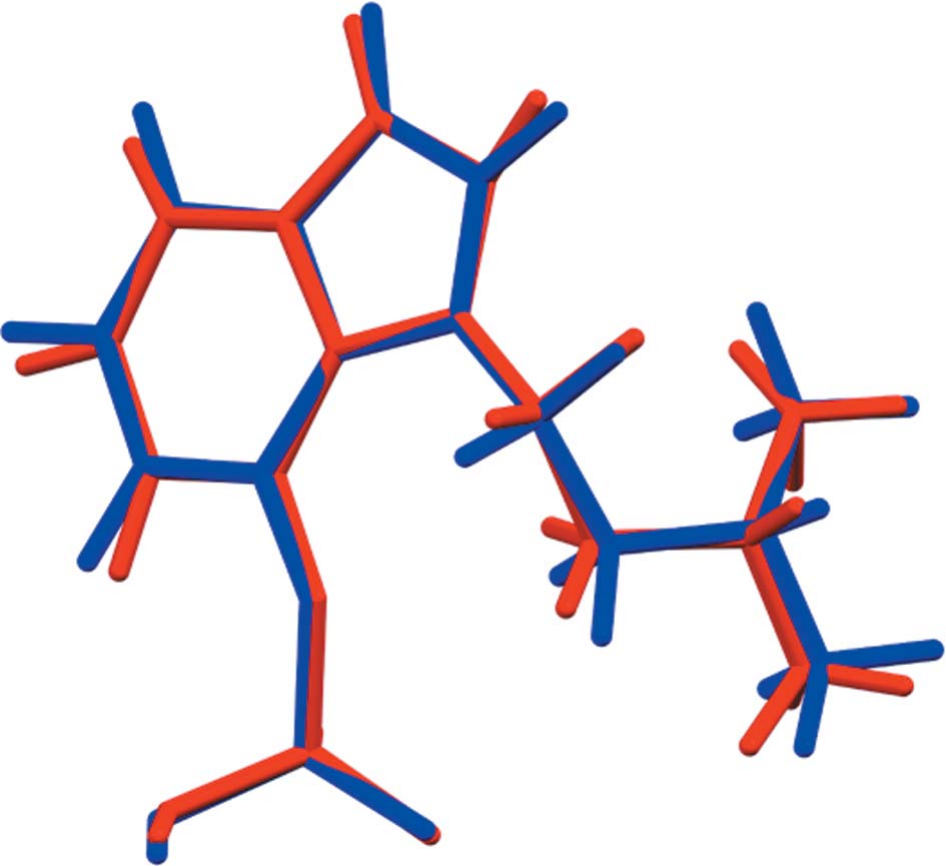
Comparison of the Rietveld-refined (red) and VASP-optimized (blue) structures of psilocybin Polymorph B Mol­ecule 2. The r.m.s. Cartesian displacement is 0.121 Å.

**Figure 8:**
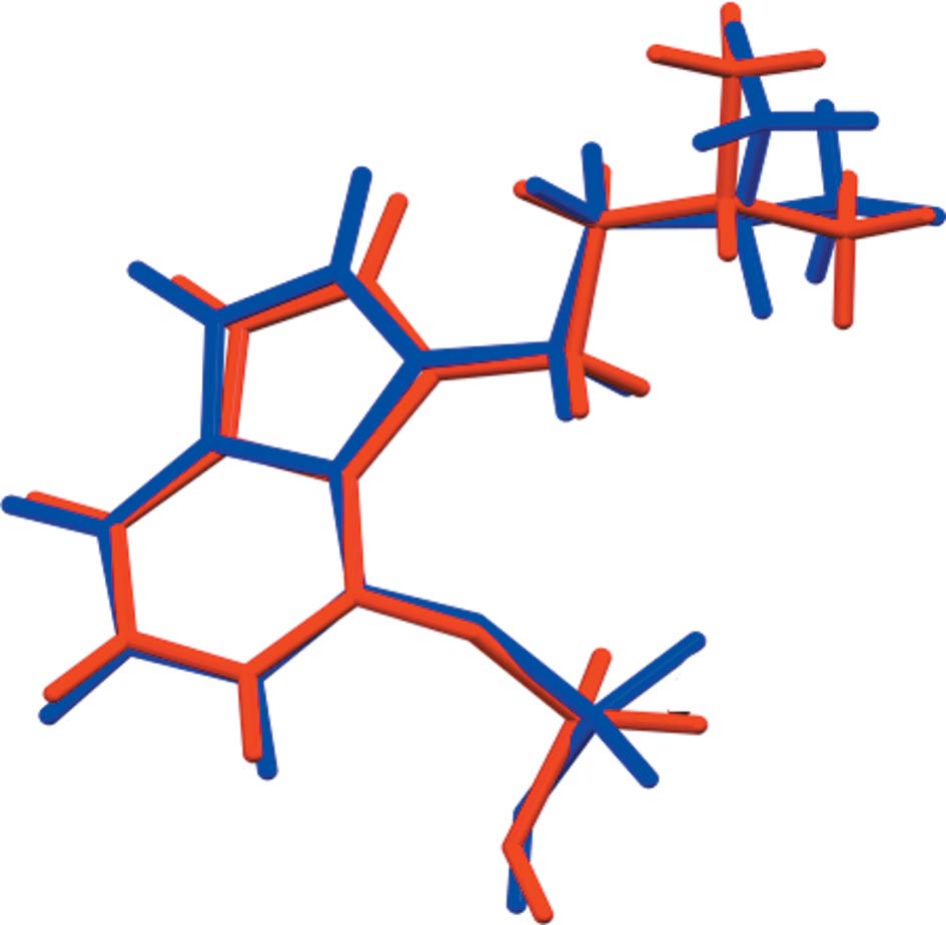
Comparison of the Rietveld-refined (red) and VASP-optimized (blue) structures of psilocybin Hydrate A (OKOKAD). The r.m.s. Cartesian displacement is 0.480 Å.

**Figure 9:**
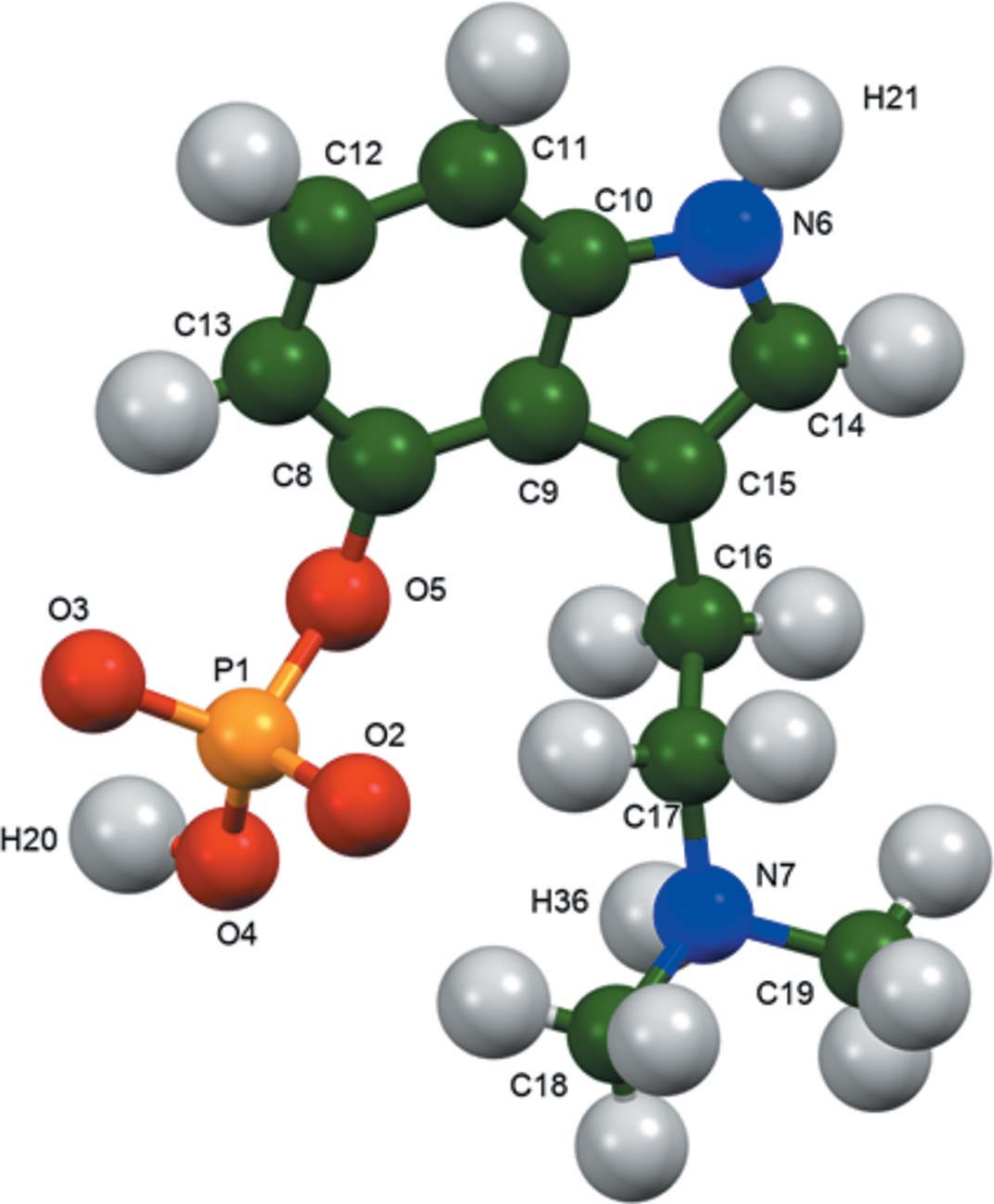
The asymmetric unit of psilocybin Polymorph A, with the atom numbering. The atoms are represented by 50% probability spheroids.

**Figure 10:**
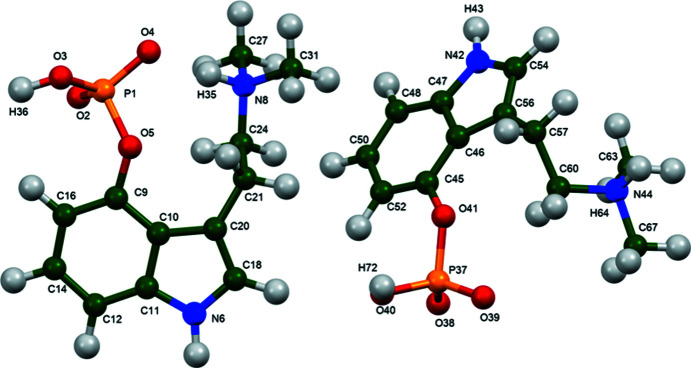
The asymmetric unit of psilocybin Polymorph B, with the atom numbering. The atoms are represented by 50% probability spheroids (fixed *U*_iso_ values).

**Figure 11:**
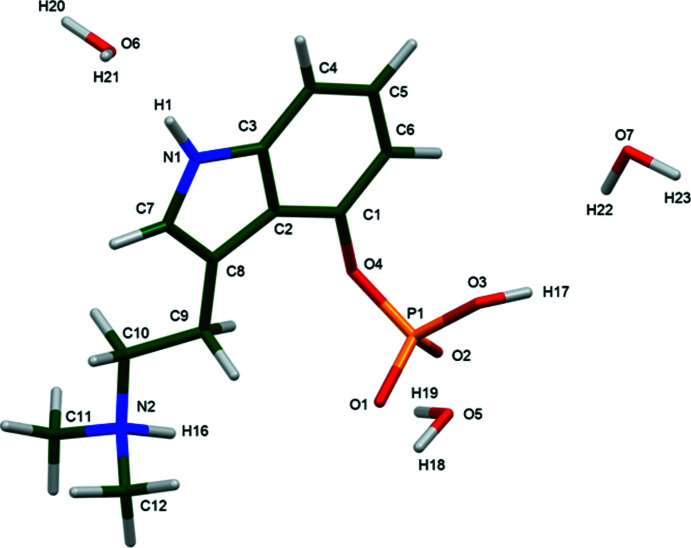
The asymmetric unit of psilocybin Hydrate A (OKOKAD), with the atom numbering.

**Figure 12:**
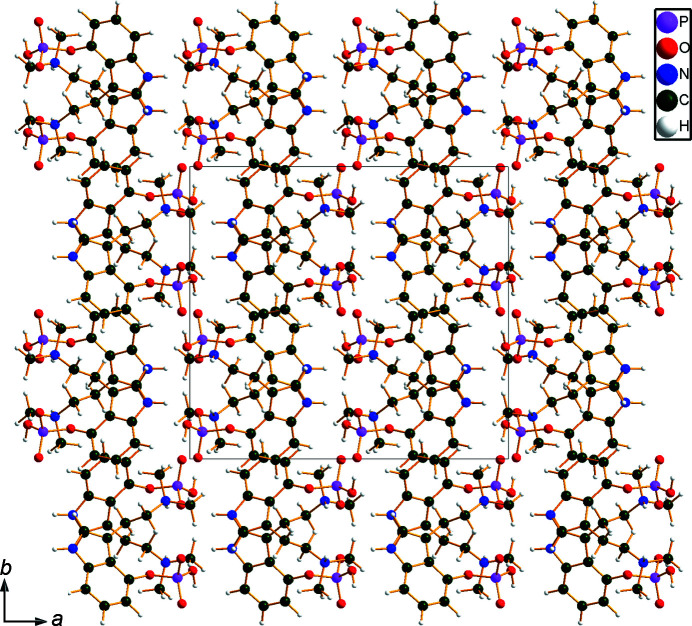
The crystal structure of psilocybin Polymorph A, viewed down the *c* axis.

The r.m.s. Cartesian displacement of the non-H atoms in the Rietveld-refined and DFT-optimized structures of Form A is 0.053 Å (Fig. 5 ▸). The com­parable qu­anti­ties for the two independent psilocybin mol­ecules in Form B are 0.160 (Fig. 6 ▸) and 0.121 Å (Fig. 7 ▸). These values are well within the normal range for correct structures (van de Streek & Neumann, 2014 ▸), and provide evidence that the structures are correct. The r.m.s. displacement for the trihydrate OKOKAD structure is 0.480 Å (Fig. 8 ▸), which is outside the normal range for correct single-crystal structures (van de Streek & Neumann, 2010 ▸). The major differences lie in the di­methyl­ammonium portion of the mol­ecule. All of the bond distances, angles, and torsion angles in the experimental single-crystal structure fall within the normal ranges, as indicated by a *Mogul* Geometry Check (Sykes *et al.*, 2011 ▸; Bruno *et al.*, 2004 ▸). In the VASP-optimized structure, the O1—P1—O4—C1 and O3—P1—O4—C1 torsion angles are flagged as unusual; these are part of a broad distribution of similar torsion angles and are not of concern. The C9—C10—N2—C11 torsion angle is also flagged as unusual; this lies on the tail of the *trans* portion of a bimodal *trans*/*gauche* distribution. The positions of water mol­ecules O5 and O7 differ significantly, however. This discussion concentrates on the DFT-optimized structures. The asymmetric unit (with atom numbering) of Form A is illustrated in Fig. 9 ▸, Form B in Fig. 10 ▸, and the trihydrate OKOKAD in Fig. 11 ▸. The crystal structures are presented in Figs. 12 ▸, 13 ▸ and 14 ▸. The VASP calculations indicated that Form B is 11.8 kJ mol^−1^ lower in energy than Form A. This energy difference is close to the expected accuracy of such calculations, so the structures must be considered similar in energy.

The crystal structures of Form A and Form B are characterized by alternating layers of hydro­carbon fragments and hydrogen bonds: parallel to the *bc* planes in Form A and the *ab* planes for Form B, reflecting the different choices of unit cells. In the trihydrate OKOKAD, the layers are more corrugated and there are pockets in which the water mol­ecules reside.

The thermal expansion of OKOKAD between 152 and 302 K is anisotropic (Table 4 ▸). The expansion is reduced along the *c* axis, which is perpendicular to the corrugated layers. The mol­ecular structures at the two tem­per­atures are essentially identical, so the expansion involves inter­molecular inter­actions.

**Figure 15:**
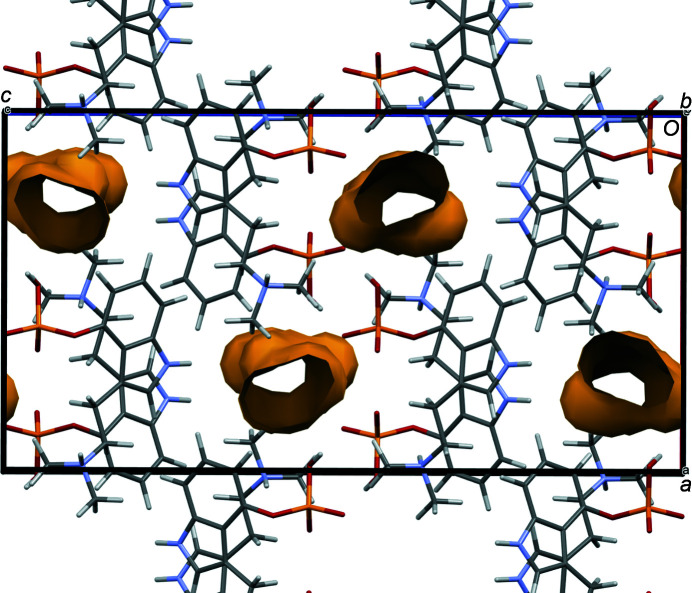
The crystal structure of psilocybin Hydrate A with the water mol­ecules removed. The probe radius is 1.2 Å.

The three water mol­ecules were removed from the OKOKAD structure, and the symmetry was lowered to *P*1 in *Materials Studio*. The structure was optimized (including the cell) using the Forcite module. A symmetry search indicated retention of the space group *Pbca*. The unit-cell parameters changed to *a* = 14.264988, *b* = 8.265617, *c* = 27.012137 Å, and *V* = 3184.97 Å^3^. The structure retains voids (Fig. 15 ▸), so the dehydration mechanism to form Polymorph A is currently unknown.

In the VASP-optimized structure of Form A, all of the bond distances and angles fall within the normal ranges, as indicated by a *Mercury Mogul* Geometry check (Macrae *et al.*, 2020 ▸). Only the C16—C17—N17—C18 torsion angle is flagged as unusual; it lies on the tail of one of the peaks of a bimodal distribution of similar torsion angles. A similar analysis of the Rietveld-refined structure indicated a few unusual bond distances (*Z*-scores 3–4) and a different unusual torsion angle in the side chain. A *Mogul* analysis of the VASP-optimized Form B structure indicated the unusual torsion angles C20—C21—C24—N8, C54—C56—C57—C40, and C56—C57—C60—N44 in the side chains. The first two lie on tails of distributions, while the third is truly unusual. *Mogul* analysis of the Rietveld-refined structure of Form B reveals (perhaps surprisingly) few unusual features, but they include torsion angles in the side chains. (All of the bond distances and angles were restrained.) *Mogul* analysis of the VASP-optimized OKOKAD structure indicates that the torsion angles O1—P1—O4—C1 and O3—P1—O4—C1 are unusual; these are part of broad distributions of similar torsion angles and are not of concern. The torsion angle C9—C10—N2—C11 is also flagged as unusual; this lies on the tail of one peak of a bimodal distribution. *Mogul* analysis of the experimental OKOKAD structure reveals no unusual intra­molecular features, but there are short inter­molecular contacts.

**Figure 16:**
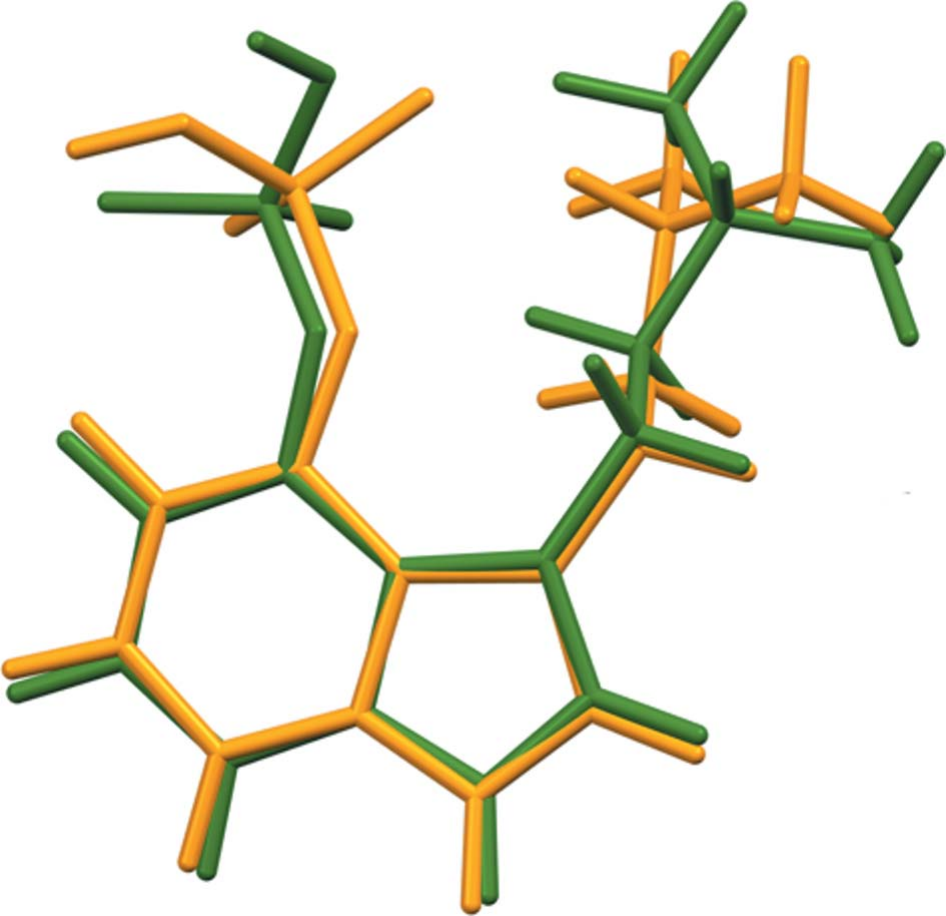
Comparison of psilocybin Polymorph A (green) and Polymorph B Mol­ecule 1 (orange).

**Figure 18:**
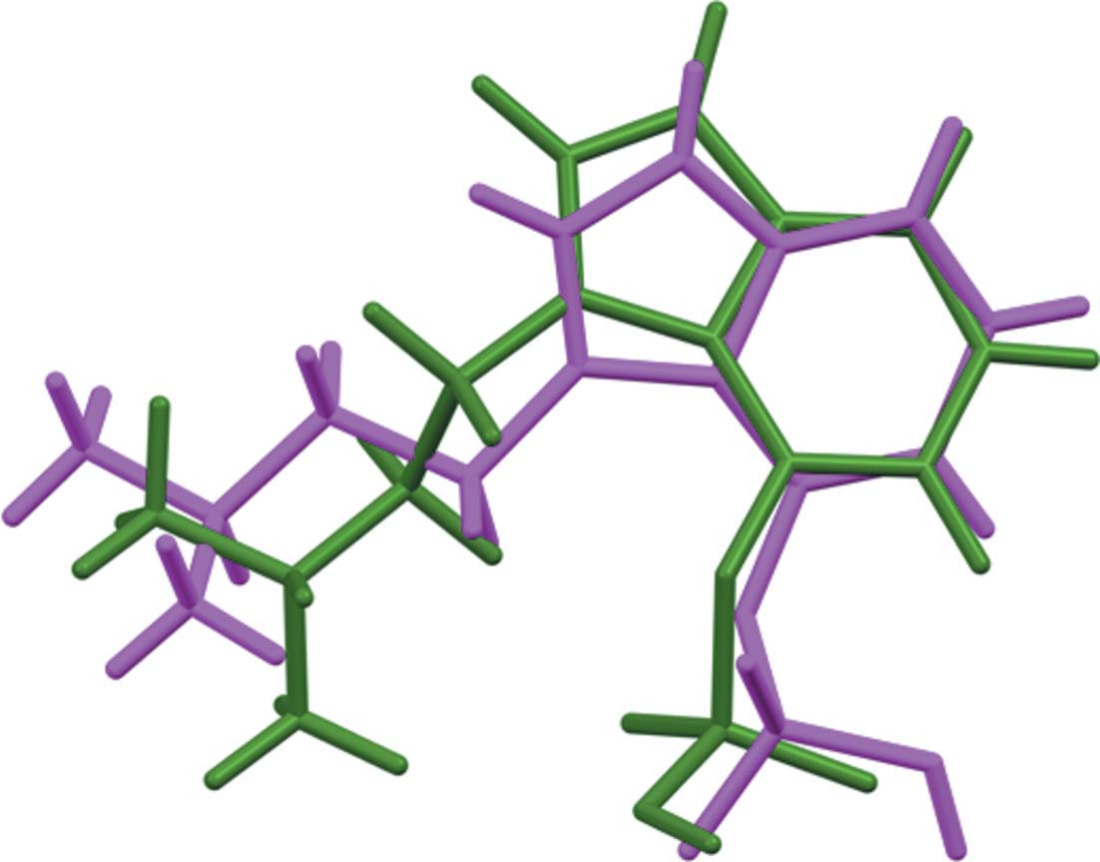
Comparison of psilocybin Hydrate A (green) and Polymorph A (magenta).

The conformations of the psilocybin mol­ecules in these structures differ. The r.m.s. Cartesian displacement of Polymorph A is 0.827 and 1.335 Å for mol­ecules 1 and 2 in Polymorph B (Figs. 16 ▸ and 17 ▸). Polymorph A differs from OKOKAD by 1.356 Å (Fig. 18 ▸) and OKOKAD differs from both mol­ecules in Polymorph B. Quantum chemical geometry optimizations of the isolated psilocybin mol­ecules in these structures (DFT/B3LYP/6-31G*/water) using *Spartan’18* (Wavefunction, 2020 ▸) indicated that mol­ecule 1 in Polymorph B has the lowest energy. Mol­ecule 2 in Polymorph B is 34.9 kJ mol^−1^ higher in energy, Polymorph A is 12.3 kJ mol^−1^ higher, and OKOKAD is 36.3 kJ mol^−1^ higher. A mol­ecular mechanics (MMFF) conformational analysis indicated that the lowest-energy conformation has an intra­molecular N^+^—H⋯OP hydrogen bond, and that mol­ecule 1 of Polymorph B is essentially in the minimum-energy conformation. Inter­molecular inter­actions are thus important in determining the solid-state conformations.

Analysis of the contributions to the total crystal energy using the Forcite module of *Materials Studio* (Dassault Systèmes, 2021 ▸) suggests that angle distortion terms dominate the intra­molecular deformation energy. The inter­molecular energy is dominated by electrostatic attractions, which in this force-field-based analysis include hydrogen bonds. The hydro­gen bonds are better analysed using the results of the DFT calculation.

The Bravais–Friedel–Donnay–Harker (Bravais, 1866 ▸; Friedel, 1907 ▸; Donnay & Harker, 1937 ▸) morphology suggests that we might expect blocky morphology for Polymorph A, and platy morphology with {001} as the major faces for Polymorph B and OKOKAD. Most of the powder samples of this material exhibited strong preferred orientation, which was modeled using spherical harmonics.

### Hydrogen bonding

As expected, hydrogen bonds are important in these structures (Tables 5 ▸, 6 ▸ and 7 ▸). In each of the three structures, the P—O—H group forms a strong inter­molecular O—H⋯O hydrogen bond to another phosphate O atom. These hydrogen bonds form rings with graph sets 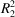 (8) (Etter, 1990 ▸; Bernstein *et al.*, 1995 ▸; Shields *et al.*, 2000 ▸). In Forms A and B, both the protonated N atoms and the ring N—H group form inter­molecular N—H⋯O hydrogen bonds to phosphate O atoms, but in Form B, one of the side chain N^+^—H⋯O hydrogen bonds is intra­molecular. In OKOKAD, both of the N—H groups act as donors to water mol­ecules. In OKOKAD, there are both water–phosphate and water–water hydrogen bonds. The energies of the O—H⋯O hydrogen bonds were calculated using the correlation of Rammohan & Kaduk (2018 ▸), and the energies of the N—H⋯O hydrogen bonds were calculated using the correlation of Wheatley & Kaduk (2019 ▸). Many C—H⋯O hydrogen bonds also contribute to the lattice energies.

### Qu­anti­tative Phase Analysis (QPA) for various psilocybin samples

Knowledge of the structures for psilocybin Hydrate A, Polymorph A, and Polymorph B made possible the application of RM-based QPA on available PXRD patterns of psilocybin from various sources to estimate the amounts of each form present (Table 8 ▸).

## Discussion

### Assessment of detected phases in bulk psilocybin

**Figure 19:**
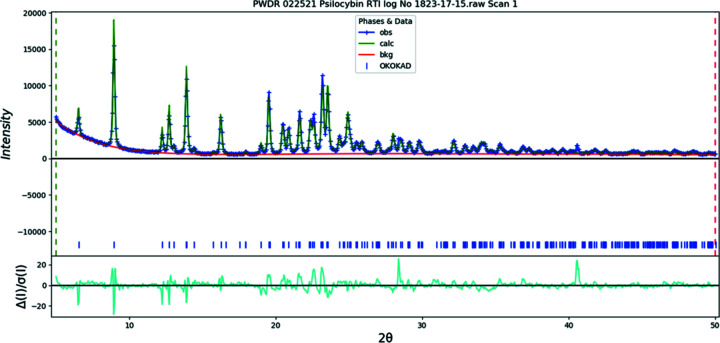
The Rietveld plot for the refinement of psilocybin Sample 1 (Hydrate A). The blue crosses represent the observed data points and the green line is the calculated pattern. The cyan curve is the normalized error plot.

Originally manufactured by Sandoz in December 1963, Sample 1 was the oldest psilocybin analyzed. PXRD data were collected in 2020, and the 2021 QPA experiment indicated that only Hydrate A was detectable from the pattern (Fig. 19 ▸). In contrast, Kuhnert-Brandstätter & Heindl (1976 ▸) noted that Sandoz psilocybin from the same time frame was anhydrous. They also noted that the anhydrate was hygroscopic and that the preferred form is the hydrate. These observations were consistent with our unpublished data, indicating that inter­conversion between Polymorph A and Hydrate A occurred at a water activity (*A*_w_) value of approximately 0.3. Taken together, evidence of the hygroscopicity of the anhydrate along with prior literature descriptions imply that Sample 1 originally existed as the anhydrous Polymorph A and/or Polymorph B, with inter­conversion to Hydrate A predicted if, over the last six decades, the bulk material was not stored under controlled low-humidity conditions. Similarly, Sample 3 was manufactured at least as early as 1976 and analyzed by PXRD in 2020. The results were analogous to Sample 1, with Hydrate A being the dominant phase present in this material. The sample vial stated that the material contained about 0.8% moisture (Fig. S2 in the supporting information), whereas the trihydrate form contains 15% water by mass. Consistent with the observations for Sample 1, the age of Sample 2, unknown storage conditions, and specified low water content, indicated that it was initially com­posed of the anhydrous polymorph(s) which inter­converted over time to Hydrate A due to water ingress. Hydrate A also appears in varying trace amounts, up to about 5%, in several samples across Table 8 ▸. These observations support the conclusion, originally presented in Kuhnert-Brandstätter & Heindl (1976 ▸), that the hydrate is the crystalline form of psilocybin that would unpreventably arise from solid material exposed to water. Nevertheless, a 2021 continuation to the Londesbrough *et al.* (2019 ▸) patent purported to claim Hydrate A as a newly discovered crystalline form and should be subject to closer investigation con­sidering the literature precedent and historical sample data described here.

While Samples 1 and 3 were characterized by modern PXRD analysis on a vintage sample of psilocybin, Sample 2 described in Folen (1975 ▸) offered the only known PXRD data collected on psilocybin contemporaneously. QPA on the Folen sample was made possible by converting the reported peak list to a pseudo-raw data file. In contrast to Samples 1 and 3, this material was characterized by PXRD reflections that were consistent primarily with Polymorph A. Both Polymorph B and Hydrate A were also detectible; however, given the low resolution of the PXRD data and processing required to make it amenable to QPA by RM, the results reported here should not be considered precise estimates for the relative concentrations of the two minor forms. Nevertheless, this modern inter­pretation of the results in Folen indicates that the three predominant crystalline forms of psilocybin existed and were described as early as 1975, and that variable amounts of these three phases could be expected in historical samples of bulk psilocybin. When this modern inter­pretation of Folen is taken together with the other evidence of inter­conversion and widespread synthesis of psilocybin by various researchers, mixtures of psilocybin Polymorph A, Polymorph B, and Hydrate A are seen as inevitable.

Sample 4 was retrieved from the Johns Hopkins University School of Medicine Clinical Pharmacy, where it had been stored under humidity-controlled conditions. QPA on PXRD patterns for this sample indicated that it consisted of anhydrate Polymorph A with possibly a trace amount of Hydrate A. Sample 4 represented bulk psilocybin that was available to multiple universities in support of at least four separate clinical trials conducted between 2015 and 2018. Those clinical trials, in combination with the QPA results for Sample 4, establish precedent for the administration of psilocybin Polymorph A to human patients in a controlled clinical setting at least as early as the year 2015.

**Figure 20:**
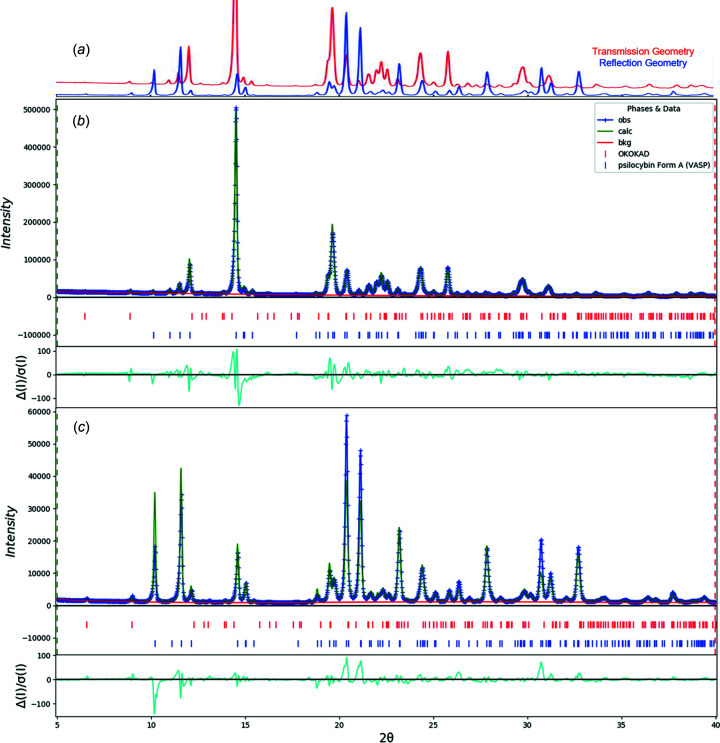
(*a*) Comparison of PXRD patterns for Sample 4 collected by transmission geometry (red) and reflection-based configurations (blue). (*b*) The Rietveld plot for the refinement of psilocybin Sample 4 (Hydrate A and Polymorph A) using transmission data. The blue crosses represent the observed data points and the green line is the calculated pattern. The cyan curve is the normalized error plot obtained by transmission geometry. (*c*) The Rietveld plot for the refinement of psilocybin Sample 4 (Hydrate A and Polymorph A) using reflection data. The blue crosses represent the observed data points and the green line is the calculated pattern. The cyan curve is the normalized error plot.

To demonstrate the variability in peak intensity for powder patterns from Polymorph A, Sample 4 was analyzed by PXRD using both transmission and reflection-based configurations. Given the needle-like morphology of Polymorph A, preferred orientation effects had a significant influence on the appearance of the two patterns when the samples were prepared by packed capillary *versus* reflection geometries (Fig. 20 ▸). Modeling the preferred orientation using 8th- and 4th-order spherical harmonics for the reflection and transmission patterns, respectively, provided com­parable results for QPA on the two patterns, though the pattern obtained by transmission geometry provided higher-quality data given the difference in texture index calculated for the two patterns: 8.25 for the reflection pattern and 1.25 for the transmission pattern. The pole figure for the reflection sample is consistent with the expected <001> needles lying parallel to the specimen plane.

**Figure 21:**
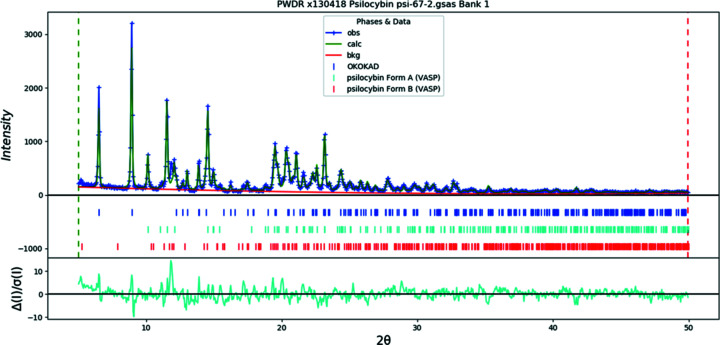
The Rietveld plot for the refinement of psilocybin Sample 5 (Hydrate A, Polymorph A, and Polymorph B). The blue crosses represent the observed data points and the green line is the calculated pattern. The cyan curve is the normalized error plot.

Samples 5–7 all originate from the same facility and were obtained using similar crystallization conditions. Like Sample 2, the Rietveld plot for Sample 5 (Fig. 21 ▸) indicated significantly detectible amounts of all three forms, with Polymorph A being the dominant form (Table 8 ▸) and approximately 12% Polymorph B present. In contrast, Sample 6 was com­posed entirely of Hydrate A, while Sample 7 consisted of Polymorph A with an insignificant trace of Hydrate A. The QPA results for these three samples highlight the potential variability in the preferred polymorphs of bulk psilocybin produced by aqueous crystallization, followed by heated vacuum drying. Together with Sample 4, Samples 6 and 7 represented the psilocybin API that was used by research universities in support of human clinical trials and further establish precedent for the use of both psilocybin Hydrate A and Polymorph A, respectively, as early as 2017 (Brown *et al.*, 2017 ▸; Nicholas *et al.*, 2018 ▸).

**Figure 22:**
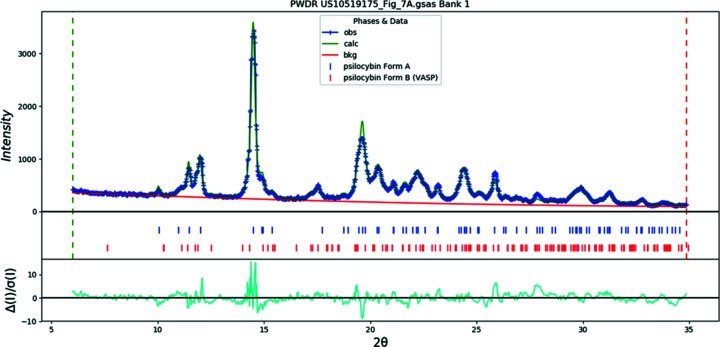
The Rietveld plot for the refinement of psilocybin Sample 8 (Polymorph A and Polymorph B), indicating that the material described as *Polymorph A* in Londesbrough *et al.* (2019 ▸) consisted of a mixture of these two phases. The blue crosses represent the observed data points and the green line is the calculated pattern. The cyan curve is the normalized error plot.

**Figure 23:**
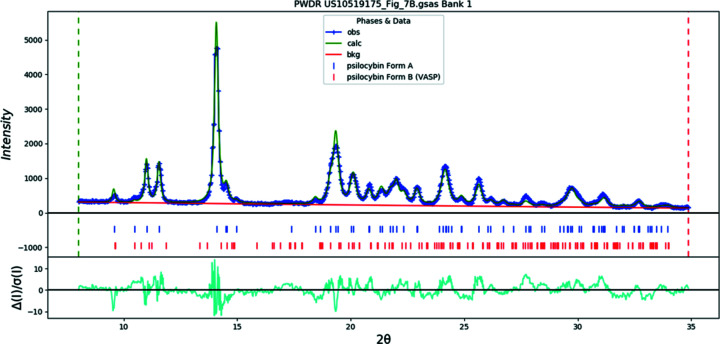
The Rietveld plot for the refinement of psilocybin Sample 9 (Polymorph A and Polymorph B). The blue crosses represent the observed data points and the green line is the calculated pattern. The cyan curve is the normalized error plot.

Samples 8–11 were obtained from scans of the provided diffractograms in Londesbrough *et al.* (2019 ▸) and converted to pseudo-raw data files allowing for analysis by QPA. In support of the hypothesis proposed in §1 and in opposition to the ‘isostructural variant’ assertion, QPA results for Sample 8 (labeled *Polymorph A* in the patent application) indicated that this material consisted of a mixture of both Polymorph A (as labeled in this publication) and Polymorph B phases. The approximate ratio of Polymorph A to Polymorph B was 81:19. The Rietveld plot for Sample 8 (Fig. 22 ▸) clearly indicates that the perturbation at 17.5° 2θ, which the authors identified as the distinguishing feature of the material, was a reflection contributed by Polymorph B. The Rietveld plot for Sample 9 (*Polymorph A′* in the patent application) (Fig. 23 ▸) also indicates the presence of a trace amount of Polymorph B, suggesting that single-phase Polymorph A was never achieved by the inventors. For com­parison, QPA was also performed on the patterns for Hydrate A and Polymorph B depicted in Londesbrough *et al.* (2019 ▸) and appear in Table 8 ▸ as Samples 10 and 11, respectively.

Subsequent patents include additional dependent claims defining *Polymorph A versus Polymorph A′* to describe the presence of a reflection at 10.1° 2θ in *Polymorph A′* where this reflection was ‘absent or substanti­ally absent’ in *Polymorph A*. We note from the solved structure for Polymorph A that this reflection corresponds to indices [200], and its intensity would be particularly sensitive to preferred orientation effects, as illustrated by com­parison of the PXRD patterns for Samples 4a and 4b analysed by both reflection and transmission geometry [Fig. 20 ▸(*a*)]. The transmission Sample 4a, which was less impacted by preferred orientation, displayed minimal intensity of the 10.1° 2θ peak, whereas the reflection geometry sample provided a strong peak at 10.1° 2θ. Therefore, the variability in observed intensity of the 10.1° 2θ reflection would be correlated with PXRD sample preparation conditions.

Samples 12–18 were obtained from chemistry process development lots and are differentiated by the ratios of acetone and water used in the recrystallization and also the time spent drying *in vacuo* (Table 3 ▸) following isolation of the solid. Except for Sample 17, which was dried at 38 °C, all others were maintained at a nominal tem­per­ature of 40 °C during drying for 1–4 d. QPA on these samples indicated that they were all representative of Polymorph A with an insignificant trace of Hydrate A in several samples. Samples 13–18 provided examples to indicate that recrystallization of psilocybin from a mixture of acetone and water followed by drying *in vacuo* could provide Polymorph A, whereas all other samples tested were crystallized from water directly. Consistent with the findings reported in Kuhnert-Brandstätter & Heindl (1976 ▸), psilocybin crystallized from organic solvents containing water were found to provide Hydrate A.

QPA of Sample 19 indicated that it consisted of Polymorph A exclusively, with Hydrate A and Polymorph B undetectable. The sample was collected from a 1.2 kg scale crystallization and provided evidence in contrast to the assertion in Londesbrough *et al.* (2019 ▸) that the crystalline habit of psilocybin was not scale dependent. That is, the additional reflection at 17.5° 2θ was not correlated with psilocybin produced at large scale in the case of Sample 19. Finally, Sample 20 provided the reference pattern for Hydrate A and Sample 21 provided the reference pattern for Polymorph B (Fig. 1 ▸), and were essentially single phase for each respective form.

**Figure 24:**
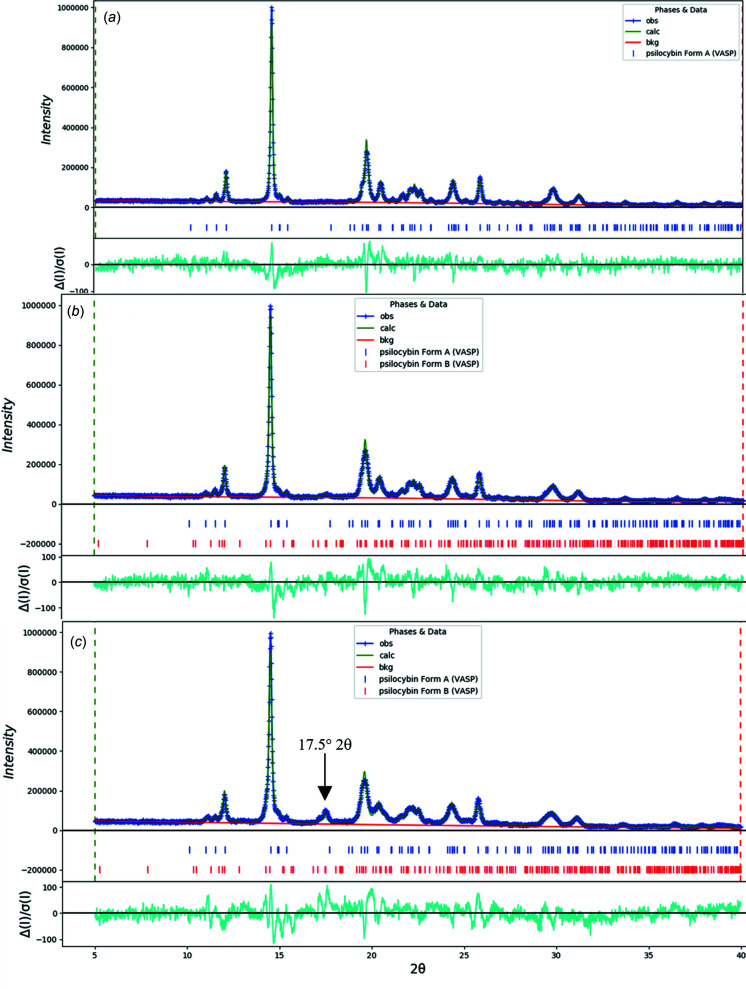
(*a*) The Rietveld plot for the refinement of psilocybin Sample 22 (Hydrate A, heated 25 h at 40 °C). The blue crosses represent the observed data points and the green line is the calculated pattern. The cyan curve is the normalized error plot. (*b*) The Rietveld plot for the refinement of psilocybin Sample 23 (Hydrate A, heated 25 h at 45 °C). The blue crosses represent the observed data points and the green line is the calculated pattern. The cyan curve is the normalized error plot. (*c*) The Rietveld plot for the refinement of psilocybin Sample 24 (Hydrate A, heated 25 h at 55 °C). The blue crosses represent the observed data points and the green line is the calculated pattern. The cyan curve is the normalized error plot.

Samples 22–24 supported an experiment to better inform large-scale API drying campaigns and explore the thermal relationship between the inter­conversion behavior of Polymorph A and Polymorph B during the dehydration of Hydrate A. Psilocybin Hydrate A was incubated at different tem­per­atures for 25 h in an open vial at atmospheric pressure without vacuum and PXRD data were subsequently collected on each sample (Fig. 24 ▸). Sample 22, which was incubated at 40 °C, provided Polymorph A exclusively. Sample 23 was incubated at 45 °C and was found to have converted to a mixture of Polymorph A with 8% Polymorph B present. Finally, Sample 24, held at 55 °C, followed a similar trend and provided a mixture of 77% Polymorph A and 23% Polymorph B. This experiment shows the likely origin of the variable levels of Polymorph B detected in several samples across Table 8 ▸, none of which were heated to 170 °C, *i.e.* the conditions found to provide the single-phase Polymorph B reference patterns (Samples 11 and 21). In contrast to Samples 23 and 24, which contained both Polymorph A and Polymorph B, large-scale Sample 18 was produced by first dehydrating Hydrate A under vacuum at a tem­per­ature less than 45 °C for 24 h followed by holding at 50–60 °C for an additional 24 h to remove any traces of water; these conditions resulted in single-phase Polymorph A. Samples 23 and 24 were initially dehydrated at higher tem­per­atures without vacuum and were marked by increasing amounts of Polymorph B; in contrast, both Samples 18 and 22 were initially dehydrated at 40 °C or below and provided Polymorph A exclusively. While single-phase Polymorph B (Samples 11 and 21) reference patterns were produced by thermal cycling of Polymorph A to 160–175 °C for a short amount of time, samples containing both Polymorph A and Polymorph B (*i.e.* 5, 8, and 23–24) were initially converted from Hydrate A at lower tem­per­atures. These observations indicated that the inter­conversion to Polymorph B may also occur through an alternate process during the initial collapse of the hydrate at tem­per­atures as low as 45 °C, and that slower dehydration of Hydrate A (*e.g.* without vacuum or on a large scale with a less exposed surface area crystal bed) at tem­per­atures >45 °C may also result in the formation of Polymorph B.

According to the process description in Londesbrough *et al.* (2019 ▸), the isolated Hydrate A crystals (94 g scale) were washed with water and then dried at 50 °C for 30 h to provide the diffractogram for Sample 8 (Fig. 22 ▸) consisting of Polymorph A with approximately 19% Polymorph B present. The result for Sample 8 was analogous to that of Samples 23 and 24 [Figs. 24 ▸(*b*) and 24(*c*)], where dehydration at a tem­per­ature greater than 45 °C provided mixtures of Polymorph A with up to 23% Polymorph B. Solid API volume increases in larger scale drying processes, as in the example described by Londesbrough *et al.* (2019 ▸), and typically results in a thicker bulk crystal bed depth relative to exposed surface area. Consequently, dehydration efficiency becomes limited by the dimensions of the drying trays and the vacuum oven utilized. In the case of psilocybin, the dehydration rate of Hydrate A would therefore decrease proportionally as scale is increased. As a result, the collapse of Hydrate A may have occurred more slowly than expected in Sample 8 *versus* Sample 9 (the small-scale example) from Londesbrough *et al.* (2019 ▸) to provide a greater opportunity for the hypothetical alternate low-tem­per­ature inter­conversion pathway to Polymorph B to occur. In summary, new observations indicate that rapid dehydration of psilocybin Hydrate A at lower tem­per­atures and reduced pressure tended to form single-phase Polymorph A, whereas slower dehydration at higher tem­per­atures formed both Polymorph A and Polymorph B. These observations inform the potential correlations between reports on psilocybin batch scale leading to the appearance of 17.5° 2θ reflections in Polymorph A PXRD patterns.

The QPA results shown in Table 8 ▸ indicate that one or more of the most frequently encountered crystalline forms of psilocybin synthesized and crystallized by routine and published methods were Hydrate A, Polymorph A, and/or Polymorph B. Inspection of the Rietveld plots for each sample indicated that no other crystalline forms were readily detectable. The older examples, Samples 1–7, demonstrated the existence of bulk psilocybin represented by either Hydrate A (Samples 1, 2, and 6), Polymorph A (Samples 4 and 7), or mixed-phase materials containing all three forms (Samples 2 and 5). These samples establish that the crystalline forms claimed in the Londesbrough *et al.* (2019 ▸) patent application and continuations had already been produced through published synthetic methods and routine techniques, and were available years before their purported invention. The QPA by RM also shed light on what was described as an unexpected result in the same patent application by providing com­pelling evidence that a phase impurity, Polymorph B, was responsible for the minor PXRD reflection at 17.5° 2θ observed from psilocybin produced in large-scale batches. The hypothesis was further explored by a controlled dehydration experiment showing that Polymorph B could be controllably introduced alongside Polymorph A by slow dehydration of Hydrate A at elevated tem­per­atures. To produce large-scale batches of crystalline psilocybin as pure polymorph A, the anhydrous polymorph most commonly produced historically, we recommend observing careful control of crystal bed depth, tem­per­ature, and pressure during the dehydration of Hydrate A; systematic studies of such conditions will be the topic of subsequent publications.

## Conclusion

Structure determination and refinement from laboratory and synchrotron PXRD patterns was accom­plished for two anhydrous crystalline forms of psilocybin commonly encountered in API process development. The new structure solutions add to three previously solved forms of psilocybin. Phase estimation of the different forms in bulk psilocybin was accom­plished using the solved structures with whole pattern fitting using the Rietveld method. The analysis indicated that the three most commonly encountered crystalline forms of psilocybin obtained from routine synthesis were a trihydrate (Hydrate A), an anhydrate (Polymorph A), and a second anhydrate (Polymorph B), and that one or more of the three phases were identified in all 24 psilocybin samples evaluated, which were produced between 1963 and 2021. Recent analyses on older samples of psilocybin often revealed Hydrate A due to hygroscopicity and inter­conversion, while more recently produced samples, as well as an older sample stored under controlled humidity, were found to contain primarily Polymorph A with variable low-level amounts of Polymorph B and Hydrate A in some cases. This article provides insight on the crystalline behavior of psilocybin when produced under conditions typical in API preparation, as well as the impact of humidity during storage. Finally, the PXRD and phase analysis of historical samples offers confidence that the three most pharmaceutically relevant crystalline forms of psilocybin have been created and observed repeatedly and consistently since 1959, when the first synthesis of psilocybin was reported.

## Supplementary Material

Crystal structure: contains datablock(s) Form_A, Form_B, Form_B_VASP, OKOKAD_VASP_loT.cif, global, Form_A_VASP. DOI: 10.1107/S2053229621013164/wp3022sup1.cif

Additional figures and CIF descriptors. DOI: 10.1107/S2053229621013164/wp3022sup2.pdf

CCDC references: 2128419, 2128420, 2128421, 2128422, 2128423

## Figures and Tables

**Figure 13:**
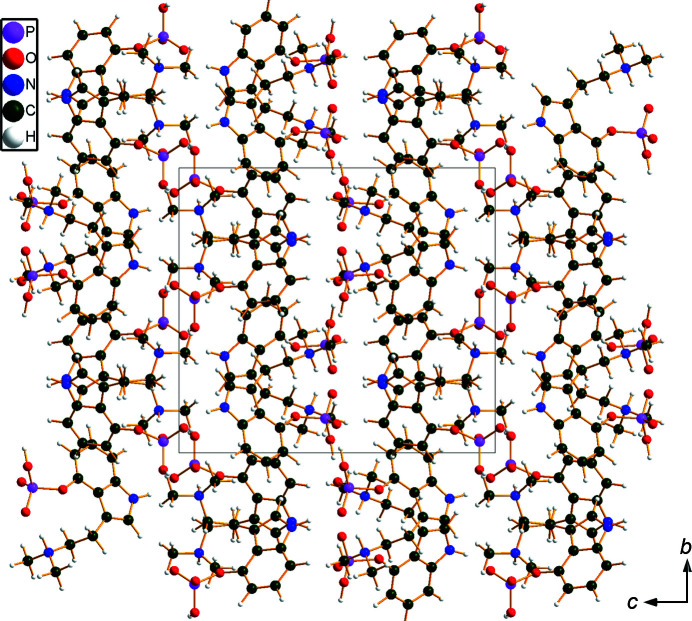
The crystal structure of psilocybin Polymorph B, viewed down the *a* axis.

**Figure 14:**
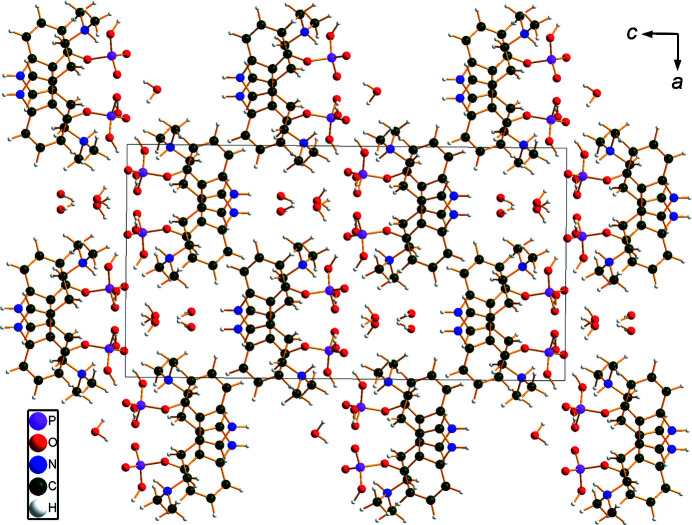
The crystal structure of psilocybin Hydrate A (OKOKAD), viewed down the *b* axis.

**Figure 17:**
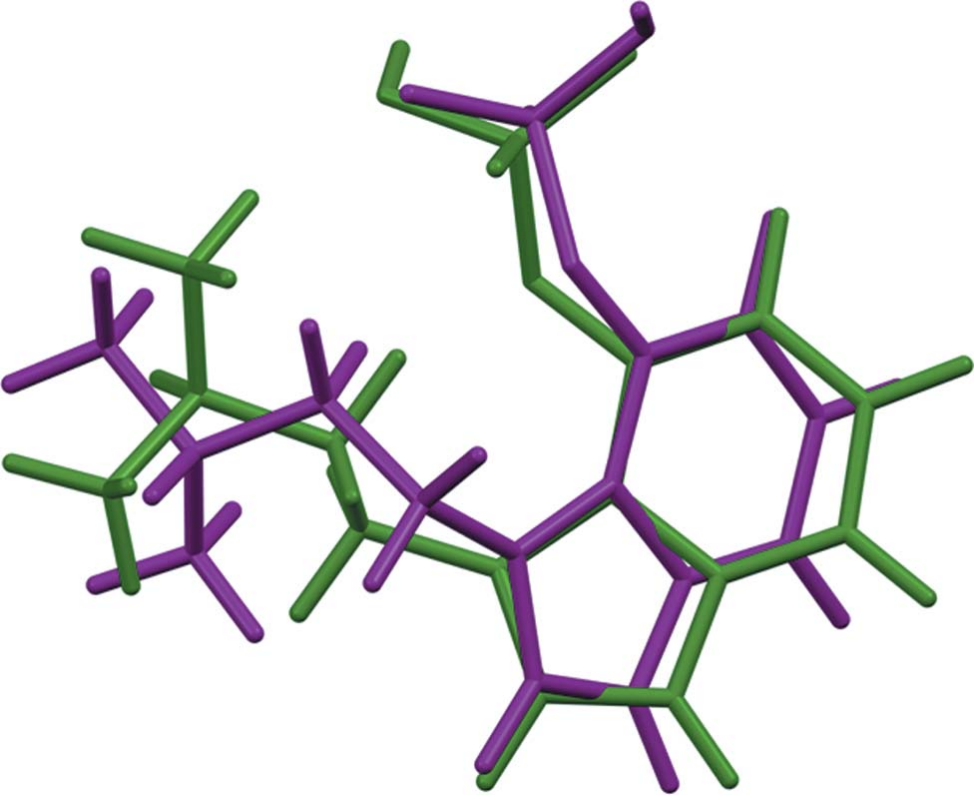
Comparison of psilocybin Polymorph A (green) and Polymorph B Mol­ecule 2 (purple).

**Table 1:** Experimental details

	Hydrate A	Polymorph A	Polymorph B
Crystal data			
Chemical formula	C_12_H_17_N_2_O_4_P·3H_2_O	C_12_H_17_N_2_O_4_P	C_12_H_17_N_2_O_4_P
*M*_r_	338.30	284.11	284.11
Crystal system, space group	Ortho­rhom­bic, *Pbca*	Ortho­rhom­bic, *Pbca*	Monoclinic, *P*2_1_/*a*
Tem­per­ature (K)	152	295	300
*a*, *b*, *c* (Å)	14.3906 (2), 8.26729 (9), 27.1581 (3)	17.4979 (2), 16.0914 (1), 9.34815 (7)	10.3986 (13), 15.4823 (19), 17.2085 (26)
α, β, γ (°)	90, 90, 90	90, 90, 90	90, 95.600 (8), 90
*V* (Å^3^)	3231.04 (6)	2632.11 (3)	2757.3 (8)
*Z*	8	8	8
Radiation type	Cu *K*α	Synchrotron	Cu *K*α
Wavelength (Å)	1.5405929/1.544430	0.458162	1.5405929/1.544430
μ (mm^−1^)	1.84	0.016	0.853
Crystal size (mm)	0.3 × 0.05 × 0.02	Powder	Powder
			
Data collection			
Diffractometer	SuperNova, Dual, Cu at home/near, EosS2	11-BM, APS	
Absorption correction	Multi-scan (*CrysAlis PRO*; Rigaku OD, 2019 ▸)		
*T*_min_, *T*_max_	0.725, 1.000		
No. of measured, independent and observed [*I* ≥ 2σ(*I*)] reflections	15245, 3239, 2898		
*R*_int_	0.032		
(sin θ/λ)_max_ (Å^−1^)	0.624		
			
Refinement			
*R*[*F* ^2^ > 2σ(*F* ^2^)], *wR*(*F* ^2^), *S*	0.032, 0.086, 1.04		
No. of reflections	3239		
No. of parameters	228		
H-atom treatment	H atoms treated by a mixture of independent and constrained refinement		
Δρ_max_, Δρ_min_ (e Å^−3^)	0.31, −0.42		

Computer programs: *CrysAlis PRO* (Rigaku OD, 2019 ▸), *SHELXS* (Sheldrick, 2008 ▸), *OLEX2.refine* (Bourhis *et al.*, 2015 ▸) and *OLEX2* (Dolomanov *et al.*, 2009 ▸).

**Table 2:** Descriptions of psilocybin samples analyzed by PXRD in order of date of availability (*i.e.* date of production or publication)

Code	Sample name	Source	Date of availability
1	RTI-1823-17-15	CM^2^	1963
2	Folen	LS^3^	1975
3	USP 0274-F	CM	1976
4	10415-25	CT^4^	2009
5	Ψ-67-2	PD^5^	2013
6	Ψ-81-1	CT	2013
7	Ψ-97-1	CT	2014
8	*Polymorph A* ^1^ (Compass Pathways)	P*L* ^6^	2017
9	*Polymorph A′* (Compass Pathways)	PL	2017
10	Hydrate A (Compass Pathways)	PL	2017
11	Polymorph B (Compass Pathways)	PL	2017
12	SPS5107/20/1	PD	2019
13	17/44/136G	PD	2019
14	17/44/132E	PD	2019
15	17/44/116*Z*	PD	2019
16	17/44/123*L*	PD	2019
17	800325750	PD	2019
18	800326600	PD	2019
19	ARN-19-002654 (Polymorph A ref.)	PD	2019
20	CG002E-004-01 (Hydrate A ref.)	PD	2019
21	CG-0019E-038-03 (Polymorph B ref.)	PD	2019
22	PL005E-004-40C	PD	2021
23	PL005E-004-45C	PD	2021
24	PL005E-004-55C	PD	2021

Notes: (1) Polymorph A as depicted in Londesbrough *et al.* (2019 ▸) and not this manuscript; (2) CM = commercial; (3) LS = literature source; (4) CT = clinical trial; (5) PD = process development; (6) PL = patent literature.

**Table 3:** Crystallization and drying conditions for Samples 12–18

Sample code	Crystallization solvent (water:acetone)	Drying tem­per­ature (°C)	Drying time under vacuum (days)
12	100:0	40	2
13	40:60	40	2
14	48:52	40	2
15	40:60	40	3
16	40:60	40	1
17	40:60	38	2
18	40:60	40	4

**Table 4:** OKOKAD lattice parameters (space group *Pbca*) The DFT-D structure optimization shrinks the unit-cell considerably and the changes are anisotropic.

	152 K/Single crystal	302 K/Powder/ratio	DFT-D 152 K/DFT-D ratio
*a* (Å)	14.39061 (16)	14.5051 (6)/1.0079	14.394189/1.00025
*b* (Å)	8.26729 (9)	8.3364 (3)/1.0083	7.950375/0.96167
*c* (Å)	27.1581 (3)	27.2314 (13)/1.0027	25.283444/0.93097
*V* (Å^3^)	3231.04	3292.84 (32)/1.0191	2893.417/0.89551

**Table 5:** Hydrogen-bond (*CRYSTAL17*) geometry (Å, °) in psilocybin Polymorph A

Hydrogen bond	*D*—H	H⋯*A*	*D*⋯*A*	*D*—H⋯*A*	Overlap (*e*)	*E* (kcal mol^−1^)
O4—H20⋯O3	1.057	1.454	2.511	178.6	0.089	16.3
N7—H36⋯O2	1.058	1.751	2.734	152.5	0.078	6.4
N6—H21⋯O2	1.031	1.899	2.905	164.2	0.061	5.7
C17—H29⋯O2	1.096	2.256*	3.337	168.3	0.030	
C19—H33⋯O3	1.096	2.517	3.518	151.4	0.017	
C13—H24⋯O3	1.088	2.388*	3.107	122.1	0.015	
C14—H25⋯O4	1.087	2.668	3.648	149.7	0.014	
C11—H22⋯O3	1.089	2.633	3.559	142.5	0.013	
C19—H34⋯O3	1.095	2.731	3.693	146.4	0.012	
C11—H22⋯O4	1.089	2.696	3.618	142.2	0.012	
C18—H32⋯O4	1.094	2.690*	3.737	160.0	0.010	

Note: (*) intra­molecular.

**Table 6:** Hydrogen-bond (*CRYSTAL17*) geometry (Å, °) in psilocybin Polymorph B

Hydrogen bond	*D*—H	H⋯*A*	*D*⋯*A*	*D*—H⋯*A*	Overlap (*e*)	*E* (kcal mol^−1^)
O40—H72⋯O39	1.043	1.491	2.533	179.0	0.085	15.9
O3—H36⋯O2	1.063	1.423	2.485	176.6	0.089	16.3
N44—H71⋯O38	1.078	1.557	2.616	166.1	0.103	7.4
N8—H35⋯O4	1.073	1.656*	2.706	164.6	0.096	7.2
N42—H43⋯O4	1.035	1.874	2.906	174.4	0.067	6.0
N6—H7⋯O39	1.034	1.791	2.797	163.1	0.064	5.8
C67—H70⋯O38	1.097	2.376	3.404	155.5	0.025	
C48—H49⋯O2	1.092	2.545	3.552	153.0	0.018	
C18—H19⋯O40	1.088	2.761	3.793	158.3	0.014	
C12—H13⋯O41	1.091	2.707	3.725	155.1	0.014	
C50—H51⋯O5	1.090	2.593	3.683	177.0	0.013	
C27—H28⋯O4	1.096	2.715	3.605	137.9	0.013	
C27—H30⋯O3	1.095	2.401	3.166	125.6	0.010	
C67—H68⋯C18	1.097	2.871	3.954	168.9	0.010	

Note: (*) intra­molecular.

**Table 7:** Hydrogen-bond (*CRYSTAL17*) geometry (Å, °) in psilocybin Hydrate A (OKOKAD)

Hydrogen bond	*D*—H	H⋯*A*	*D*⋯*A*	*D*—H⋯*A*	Overlap (*e*)	*E* (kcal mol^−1^)
O3—H17⋯O2	1.044	1.476	2.519	176.7	0.081	15.6
N2—H16⋯O5	1.067	1.723	2.746	158.9	0.084	6.7
N1—H1⋯O6	1.028	1.982	3.006	173.9	0.057	5.5
O7—H23⋯O1	0.989	1.898	2.847	159.7	0.048	11.9
O7—H22⋯O2	0.991	1.822	2.776	160.7	0.051	12.3
O6—H21⋯O7	0.975	2.648	3.533	151.1	0.011	5.7
O6—H20⋯O7	0.999	1.743	2.730	169.0	0.058	13.2
O5—H19⋯O1	0.995	1.749	2.738	172.2	0.053	12.6
O5—H18⋯O1	0.989	1.894	2.870	168.6	0.047	11.8
C12—H14⋯O6	1.097	2.519	3.604	170.1	0.026	
C12—H15⋯O5	1.094	2.433	3.482	160.2	0.021	
C12—H13⋯O2	1.093	2.478	3.423	143.9	0.014	
C11—H10⋯O5	1.095	2.767	3.732	146.9	0.013	
C11—H10⋯O7	1.095	2.744	3.462	122.8	0.011	
C9—H7⋯O4	1.102	2.417	3.358	142.5	0.011	
C11—H12⋯C6	1.093	3.545	173.0	0.013		

**Table 8:** Relative abundances of crystalline psilocybin phases in each of the samples listed in Table 2 ▸, as obtained by Rietveld-based QPA The estimates are approximate for several samples, as the PXRD data were obtained from several different diffractometers and geometries. Rietveld plots for the refinements of Samples 1, 4^*a*^, 4^*b*^, 5, 8, 9, and 22–24 are included as Figs. 19 ▸–24 ▸ ▸ ▸ ▸ ▸.

Code	Sample name	Hydrate A (%)	Polymorph A (%)	Polymorph B (%)
1	RTI-1823-17-15	100	–	
2	Folen	4.5 (4)	85.9 (54)	9.6 (30)
3	USP 0274-F	100	–	–
4^*a*^	10415-25	0.3 (1)	99.7 (6)	–
4^*b*^	10415-25	0.2 (1)	99.8 (19)	–
5	Ψ-67-2	6.5 (1)	80.9 (22)	12.5 (10)
6	Ψ-81-1	100	–	–
7	Ψ-97-1	0.2 (1)	99.8 (17)	–
8	*Polymorph A*	–	80.9 (6)	19.1 (7)
9	*Polymorph A′*	–	99.7 (8)	0.3 (3)
10	Hydrate A	100		
11	Polymorph B	–	–	100
12	SPS5107/20/1	0.1 (1)	99.9 (10)	–
13	17/44/136G	0.1 (1)	99.1 (13)	–
14	17/44/132E	–	100.0 (11)	–
15	17/44/116*Z*	0.1 (1)	99.1 (12)	–
16	17/44/123*L*	0.2 (1)	99.8 (11)	–
17	800325750	0.2 (1)	99.8 (25)	–
18	800326600	0.2 (1)	99.8 (10)	–
19	ARN-19-002654	–	100	–
20	CG002E-035-04	100	–	–
21	CG-0019E-038-03	–	–	100
22	PL005E-004-40C	–	100	–
23	PL005E-004-45C	–	91.7 (7)	8.3 (4)
24	PL005E-004-55C	–	77.4 (8)	22.6 (5)

Notes: (*a*) Sample analyzed by PXRD with transmission geometry. (*b*) Sample analyzed by PXRD with reflection geometry.
