# Supplementary material for: Psilocybin: crystal structure solutions enable phase analysis of prior art and recently patented examples
Source: Acta Crystallogr C Struct Chem. 2022 Jan 1;78(Pt 1):36–55. doi: 10.1107/S2053229621013164 (PMC8725723; doi:10.1107/S2053229621013164)
Supplement: Supplementary file 2 [file c-78-00036-sup2.pdf]

STRUCTURAL CHEMISTRY

Volume 78 (2022)

Supporting information for article:

Psilocybin: crystal structure solutions enable phase analysis of prior art and recently patented examples

Alexander M. Sherwood, Robert B. Kargbo, Kristi W. Kaylo, Nicholas V. Cozzi, Poncho Meisenheimer and James A. Kaduk

A Crystallographic Information Format (CIF) file is provided, containing the results of the Rietveld refinements (including the raw data) and the DFT optimizations.

Form_A	The refined structure of Form A
Form_A_VASP	The VASP-optimized structure of Form A
Form_B	The refined structure of Form B
Form_B_VASP	The VASP-optimized structure of Form B
OKOKAD_VASP_loT	The VASP-optimized structure of psilocybin trihydrate, using the fixed 152K lattice parameters.

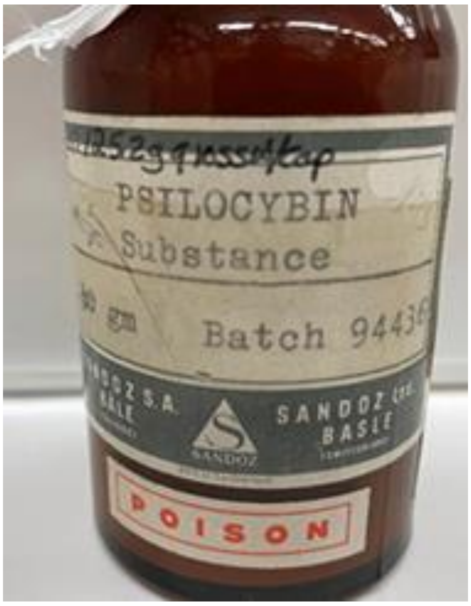

Figure S1 Sample 1 container. Printed date on bottle is “12/13/63.”

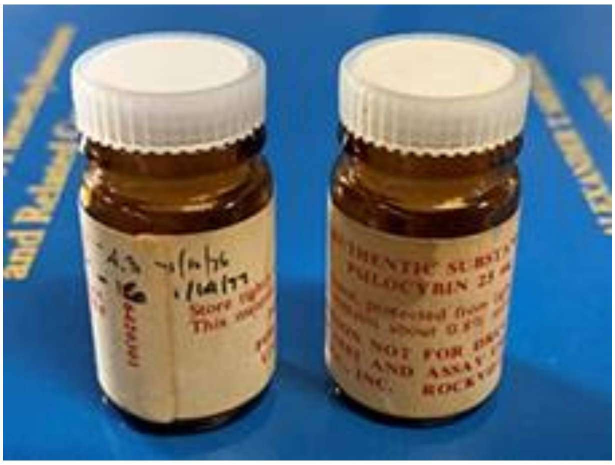

Figure S1 Sample 3 container. First record of use printed “1/10/76.” Label also states, “This material contains about 0.8% moisture.”
